# Annotation of Differential Gene Expression in Small Yellow Follicles of a Broiler-Type Strain of Taiwan Country Chickens in Response to Acute Heat Stress

**DOI:** 10.1371/journal.pone.0143418

**Published:** 2015-11-20

**Authors:** Chuen-Yu Cheng, Wei-Lin Tu, Shih-Han Wang, Pin-Chi Tang, Chih-Feng Chen, Hsin-Hsin Chen, Yen-Pai Lee, Shuen-Ei Chen, San-Yuan Huang

**Affiliations:** 1 Department of Animal Science, National Chung Hsing University, Taichung, Taiwan; 2 Agricultural Biotechnology Center, National Chung Hsing University, Taichung, Taiwan; 3 Center for the Integrative and Evolutionary Galliformes Genomics, iEGG Center, National Chung Hsing University, Taichung, Taiwan; 4 Department of Veterinary Medicine, National Chung Hsing University, Taichung, Taiwan; 5 Center of Nanoscience and Nanotechnology, National Chung Hsing University, Taichung, Taiwan; Wageningen UR Livestock Research, NETHERLANDS

## Abstract

This study investigated global gene expression in the small yellow follicles (6–8 mm diameter) of broiler-type B strain Taiwan country chickens (TCCs) in response to acute heat stress. Twelve 30-wk-old TCC hens were divided into four groups: control hens maintained at 25°C and hens subjected to 38°C acute heat stress for 2 h without recovery (H2R0), with 2-h recovery (H2R2), and with 6-h recovery (H2R6). Small yellow follicles were collected for RNA isolation and microarray analysis at the end of each time point. Results showed that 69, 51, and 76 genes were upregulated and 58, 15, 56 genes were downregulated after heat treatment of H2R0, H2R2, and H2R6, respectively, using a cutoff value of two-fold or higher. Gene ontology analysis revealed that these differentially expressed genes are associated with the biological processes of cell communication, developmental process, protein metabolic process, immune system process, and response to stimuli. Upregulation of heat shock protein 25, interleukin 6, metallopeptidase 1, and metalloproteinase 13, and downregulation of type II alpha 1 collagen, discoidin domain receptor tyrosine kinase 2, and Kruppel-like factor 2 suggested that acute heat stress induces proteolytic disintegration of the structural matrix and inflamed damage and adaptive responses of gene expression in the follicle cells. These suggestions were validated through gene expression, using quantitative real-time polymerase chain reaction. Functional annotation clarified that interleukin 6-related pathways play a critical role in regulating acute heat stress responses in the small yellow follicles of TCC hens.

## Introduction

Global warming increases environmental temperatures and affects not only humans but also livestock [1,2,3]. Animal exposure to hot environments deleteriously affects their reproductive functions. In females, heat stress adversely affects oogenesis, oocyte maturation, fertilization, and embryo development and implantation rate [4,5]. In chickens, high ambient temperatures affect their endocrine systems and reproductive and egg-laying performance [6]. Thus, in tropical areas, such as Taiwan, high temperatures and humidity during summer induce stress in poultry. The average temperature in Taiwan has increased by 0.8°C in past decades, with summer temperature and humidity reaching 38°C and 80%, respectively (http://www.cwb.gov.tw/V7/index.htm).

Approximately 12,000 oocytes are present in the ovary of a mature hen. However, only a few hundred oocytes are selected for ovulation and subsequent egg formation. A functional hen ovary contains hundreds of white cortical follicles with a diameter of 1–5 mm, small yellow follicles (SYFs) with a diameter of 6–8 mm, and large yellow preovulatory hierarchy follicles with a diameter of 9–40 mm [7,8]. The SYFs are in a crucial prehierarchical stage related to the development of follicles and the laying performance [9]. A single follicle is selected from the SYF pool every day to join the group of preovulatory follicles destined for ovulation [10,11].

The normal body temperature of chicken is 40–41°C [12]. Panting is the primary mode of heat dissipation in birds. Heat insults exceeding the capacity of bodily thermoregulation detrimentally affect production performance. Taiwan country chickens (TCCs) are native, slow-growing breeds and exhibit higher thermotolerance than do nonnative breeds [13,14]. Broiler-type B strain TCCs have been bred for body weight and comb size for over 20 generations [15]. A few reports have investigated differential gene expression in chickens in response to heat stress [13,16,17,18]; however, the effect of acute heat stress on global gene expression in the ovary, particularly in native chickens of tropical regions, has not been explored. This study thus aimed to analyze the global mRNA expression of SYF in TCCs as a basis for delineating the mechanism of acute heat stress response in chicken hens.

## Materials and Methods

### Experimental animals and management

Twelve 30-wk-old broiler-type B strain TCC hens originally bred for meat production by National Chung Hsing University [19,20] were used in this study. The care and use of all animals in the study were complied with the guidelines and was approved by the Institutional Animal Care and Use Committee of National Chung Hsing University (Taichung, Taiwan; IACUC No. 102–06). The hens, housed in individual cages at 18 wk of age, peaked in egg production at 30 weeks [15]. The hens were placed in a climate chamber for over 2 weeks for adaptation under conditions of a light:dark photoperiod of 14:10 h at 25°C and 55% relative humidity (RH) before acute heat stress treatment. Feed and water were provided ad libitum, including the acute heat stress and recovery periods.

### Conditions of acute heat stress and sample collection

After adaptation, hens were randomly allocated to four groups (three hens in each group). The control group was maintained at 25°C and 55% RH throughout the experiment. The hens in the other three groups were treated with an acute heat stress at 38°C for 2 h without recovery (H2R0), at 25°C with 2-h recovery (H2R2), and at 25°C with 6-h recovery (H2R6). The light: dark photoperiod and RH during the heat stress treatment and recovery remained the same as the adaptation period. Physiological parameters (respiratory rate and body temperature) were recorded during treatment and recovery. The respiratory rate was measured by counting the panting breaths of the chickens for 15 sec and the value was multiplied by 4 to give the number of breaths per min. The body temperature was obtained by introducing an alcohol thermometer into the cloaca of the chickens and recorded until the reading was stable. The hens were sacrificed by electric stunning and followed by bleeding from carotid artery at the end of each time point; their SYF were collected, placed overnight in cryogenic vials with 0.5 mL of RNAfter (GMbiolab Co, Ltd, Taichung, Taiwan) at 4°C, and stored at −80°C until RNA isolation. The time from sacrificing to the sample collection was limited to within 10 min.

### Gene expression analysis in response to acute heat stress through microarray analysis

A chicken 44K oligo microarray (Agilent Technologies, Santa Clara, CA, USA) was used to determine differential gene expression between the control and acute-heat -stressed groups [13]. RNA isolated from the SYF of each hen was used for reverse transcription. The second strand complementary DNA (cDNA) was synthesized from 1 μg of the total RNA and amplified using a Quick-Amp Labeling Kit (Agilent Technologies). The cDNA served as the template for in vitro transcription for producing the target cRNA in the presence of Cy3-CTP (CyDye, Agilent Technologies). In total, 1.65 μg of Cy3-labled cRNA was fragmented to an average size of approximately 50–100 nucleotides through fragmentation buffer incubation at 60°C for 30 min. Subsequently, the corresponding fragment-labeled cRNA was hybridized to the microarray at 65°C for 17 h. After washing and drying, using a nitrogen gun, the microarrays were scanned using a microarray scanner (Agilent Technologies) at 535 nm for Cy3. The scanned images were analyzed using Feature Extraction 10.5.1.1 software (Agilent Technologies) and normalized for quantifying the signal and background intensities of each feature. Data was acquired using the following criteria: (1) p < 0.01 for gene expression difference using GeneSpring software (Agilent Technologies). (2) A distinct signal from the microarray image flagged by the software. (3) A false discovery rate of < 0.05. Results of the microarray analysis were filtered from the features when flags were present or marginal in at least one of the four groups (control, H2R0, H2R2, and H2R6). The dataset of microarray analysis were submitted to Gene Expression Omnibus in the National Center for Biotechnology Information under an accession number of GSE71091.

### Gene annotation and gene network analysis of differentially expressed genes

The differentially expressed genes with known identities or with homologous sequences and functional definitions were categorized using the Gene Ontology (GO, http://www.geneontology.org/) and PANTHER (http://www.pantherdb.org/) databases according to their cellular components, biological processes, and molecular functions. Functional pathway analysis was performed using the STRING database (http://string-db.org/). Differentially expressed genes were input for generating biological networks by comparing the input list with a reference list from human databases.

### Validation of gene expressions by using quantitative real-time polymerase chain reaction

Eight differentially expressed genes that played a critical role in the annotation analysis in response to acute heat stress—heat shock protein 25 (*HSP25*); interleukin 6 (*IL6*); vitellogenin 2 (*VTG2*); metalloproteinase 13 (*MMP-13*); polymerase I and transcript release factor (*PTRF*); collagen, type II, alpha 1 (*COL2A1*); discoidin domain receptor tyrosine kinase 2 (*DDR2*); and Kruppel-like factor 2 (*KLF2*) were validated using a quantitative real-time polymerase chain reaction (qRT-PCR) analysis [13]. The sample set used in the microarray analysis was used for validation. The qRT-PCR primers and their predicted product sizes are listed in Table 1. The qRT-PCR reactions were performed on the Roche Light-Cycler Instrument 1.5 using a Light-Cycler FastStart DNA MasterPLUS SYBR Green I kit (Roche Cat. 03 515 885 001, Castle Hill, Australia). For the PCR, 2 μL of master mix, 2 μL of 0.75 mM forward and reverse primer, and 6 μL of cDNA samples were used, with each sample tested three times. The RT-PCR program was run at 95°C for 10 min, 40 cycles each at 95°C for 10 s, 60°C for 15 s, and 72°C for 10 s; subsequently, a melt curve analysis was performed. At the end of each RT-PCR run, the data were automatically analyzed by the system and an amplification plot was generated for each cDNA sample. From each of these plots, the LightCycler3 data analysis software automatically calculated the crossing point value (Cp; the crossing point corresponds to the first maximum of the second derivative curve), which was interpreted as the beginning of exponential amplification. The fold expression or repression of the target gene relative to the internal control gene, GAPDH, in each sample was calculated [13]. For consistency with the microarray analysis, the cutoff value for the differentially expressed genes was set to two-fold or higher.

**Table 1:** Primers and product size of genes used for validation using quantitative real-time polymerase chain reaction.

Gene symbol^a^	GenBank accession number	Forward (F) primers 5'-3'	Product size (base pairs)
		Reverse (R) primers 5'-3'	
*HSP25*	NM_001010842	F: CCGTCTTCTGCTGAGAGGAGTG	117
		R: ACCGTTGTTCCGTCCCATCAC	
*IL6*	NM_204628	F: AGCAAAACACCTGTTACATTTCT	96
		R: AGTCTGGCTGCTGGACATTT	
*VTG2*	NM_001031276	F: CAGCCTAACTGACAAACAGATGAAG	100
		R: GCATTCCTCATTCTCACATGAACAC	
*MMP13*	AF070478	F: TTGGTGCTAAGTATAGATGAATGCC	131
		R: TGTAGGTAGTCAGTGCTTGTTCG	
*PTRF*	NM_001001471	F: CCCTGCCTGCTAGGACAAG	149
		R: AGGTCTGGGCTCTGGAAGG	
*COL2A1*	NM_204426	F: CACTGAACGGATGGCACGAC	137
		R: CCTCCACCCGCCCTACG	
*DDR2*	CR387623	F: TGCGGACGGGAGGAACTG	103
		R: AGCAATAGGGTACTGCGAATGG	
*KLF2*	XM_418264	F:CGCCGAGGATTGGACACAG	139
		R: CACGGAGTTCACCCTTCACAG	
*GAPDH*	NM_204305	F: CATCACAGCCACACAGAAGA	122
		R: TGACTTTCCCCACAGCCTTA	

^a^ Abbreviations: *HSP25*, heat shock protein 25; *IL6*, interleukin 6; *VTG2*, vitellogenin 2; *MMP13*, metalloproteinase 13; *PTRF*, polymerase I and transcript release factor; *COL2A1*, type II alpha 1 collagen; *DDR2*, discoidin domain receptor tyrosine kinase 2; *KLF2*, Kruppel-like factor 2; *GAPDH*, glyceraldehyde-3-phosphate dehydrogenase.

### Statistical analysis

The physiological parameters of the control and heat-stressed hens during acute heat stress and recovery were analyzed using a Student *t* test in Statistical Analysis System software [21]. Multiples of changes in the microarray and qRT-PCR analysis of each individual of each group are presented as the arithmetic mean of the three replicates.

## Results

### Effect of heat stress on physiological parameters in broiler-type B strain TCCs

**Fig 1:**
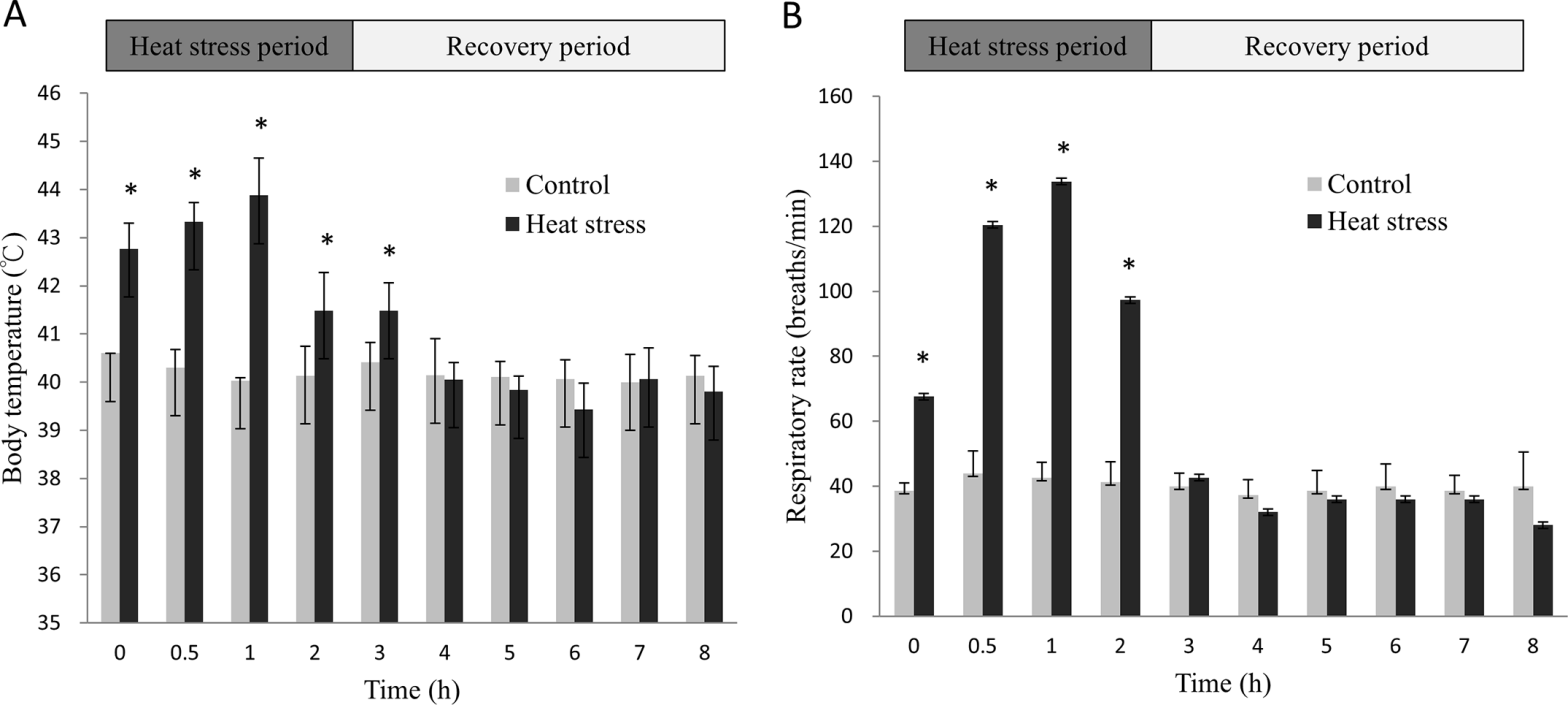
Body temperature (A) and respiratory rate (B) of acute-heat-stressed and control hens during stress and recovery periods. Data are mean ± standard error (n  =  12; n  =  6 and n  =  3 in groups with 2-h and 6-h recovery after heat stress, respectively). * Values differed between heat-stressed and control groups (p < 0.05).

To evaluate the response of hens to acute heat stress, the hens were exposed to 38°C heat stress for 2 h. The acute heat stress increased the respiratory rate and body temperature immediately after the heat treatment began (p < 0.05; Fig 1). The hens started panting 30 min after heat stress, which continued until 1 h of recovery after the heat stress. The respiratory rate and body temperature normalized during the recovery period.

### Effects of heat stress on gene expressions in the SYFs of broiler-type B strain TCCs after acute heat stress

**Fig 2:**
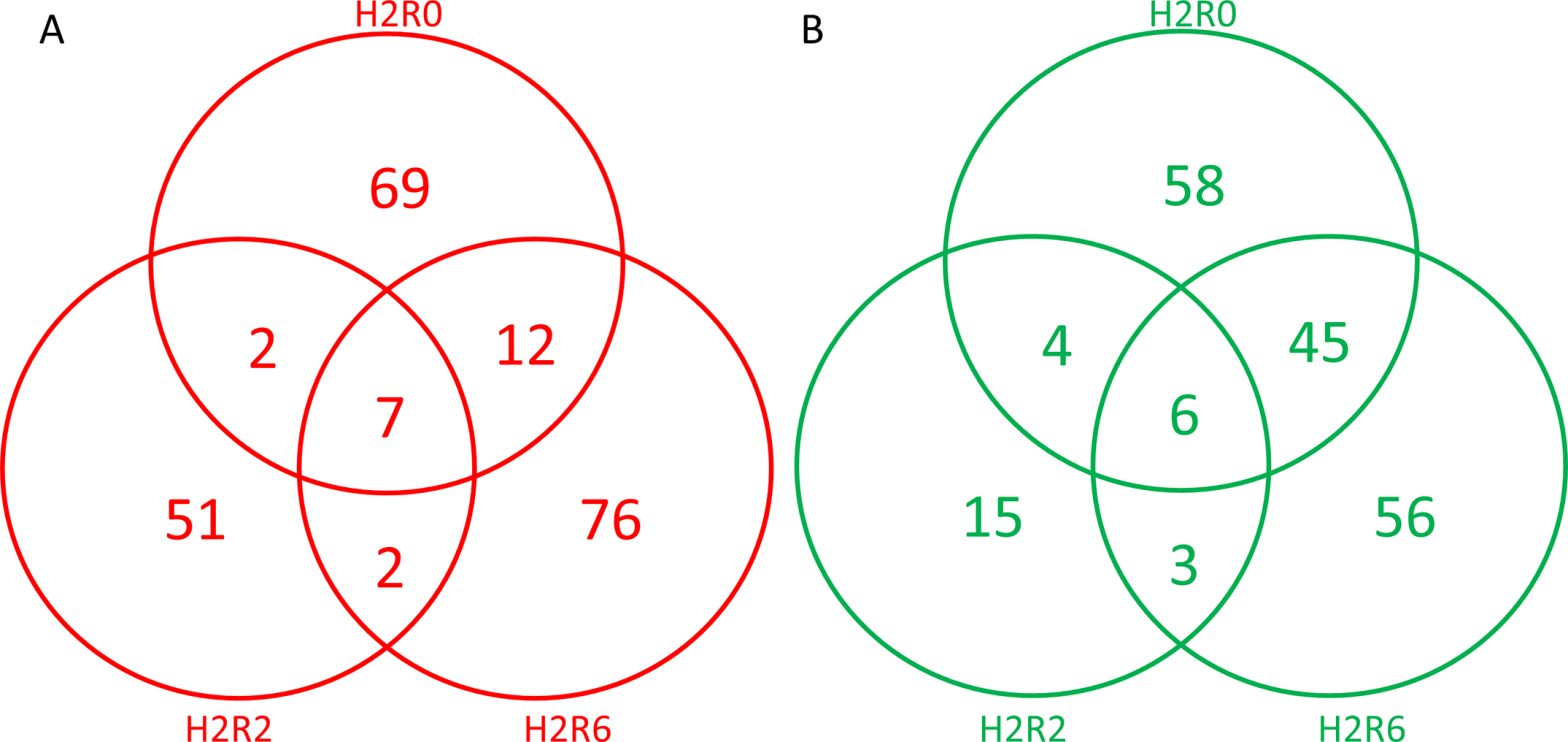
Venn diagram analysis of 219 upregulated (A) and 187 downregulated (B) genes in the small yellow follicles of broiler-type Taiwan country chickens with 38°C acute heat stress for 2 h and recovery for 0, 2, and 6 h. H2R0, recovery for 0 h after heat stress; H2R2, recovery for 2 h after heat stress; H2R6, recovery for 6 h after heat stress.

The mRNA profile of SYFs from control and heat-stressed hens were analyzed using a microarray. When using a cutoff value of a two-fold change, 406 genes showed differential expression on treatment (p < 0.05). The expression patterns of the 406 distinct genes are presented in Fig 2. Compared with the control group, the H2R0, H2R2, and H2R6 groups differed in 203, 90, and 147 genes, respectively; 69, 51, and 15 gene transcripts upregulated (S1 Table) and 58, 15, and 56 genes downregulated (S2 Table) specifically in the H2R0, H2R2, and H2R6 groups, respectively. After heat exposure, seven genes—*HSP25*, *MYOC*, *PTRF*, *RGPD1*, *SOGA3*, ChEST305c2 (*Gallus gallus* finished cDNA), and ChEST920a4 (*Gallus gallus* finished cDNA)—exhibited higher expression for all recovery times. The other six genes—*ABI3*, *GAL2*, *GAL7*, *SERPINB10*, alpha-2-macroglobulin-like 1 [ENSGALT00000023052], and ChEST478o11 (*Gallus gallus* finished cDNA)—exhibited downregulation for all recovery times.

### Functional categories of the differentially expressed genes in the SYFs of broiler-type B strain TCCs after acute heat stress

**Fig 3:**
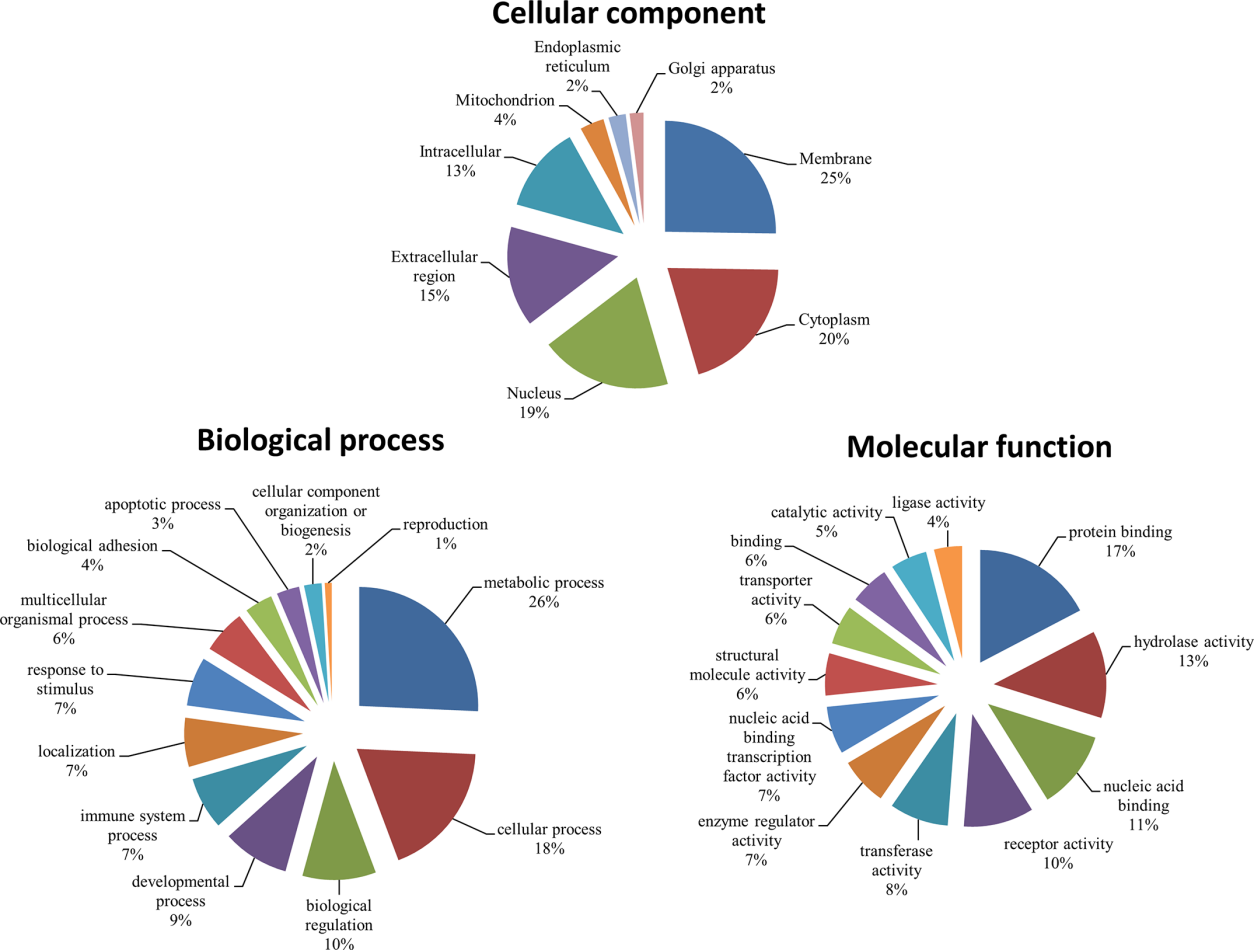
Classification of differentially expressed genes in small yellow follicles of broiler-type B strain Taiwan country chickens with 38°C acute heat stress for 2 h and recovery for 0, 2, and 6 h by cellular components (A), biological processes (B), and molecular functions (C). Only the 212 genes with known functional definitions in the Gene Ontology and PANTHER databases were included.

To characterize the functions of the differentially expressed genes, genes with known identities were subjected to GO annotation (Fig 3). The differentially expressed genes were primarily localized in the membrane, cytoplasm, nucleus, and extracellular regions. Most genes were associated with multiple biological processes and were involved in the metabolic process (26%), cellular process (18%), biological regulation (10%), developmental process (9%), immune system process (7%), localization (7%), response to stimulus (7%), and multicellular organismal process (6%). The majority of the differentially expressed genes were associated with multiple molecular functions, including protein binding (17%), hydrolase activity (13%), nucleic acid binding (11%), receptor activity (10%), transferase activity (8%), enzyme regulator activity (7%), and nucleic acid binding transcription factor activity (7%).

**Fig 4:**
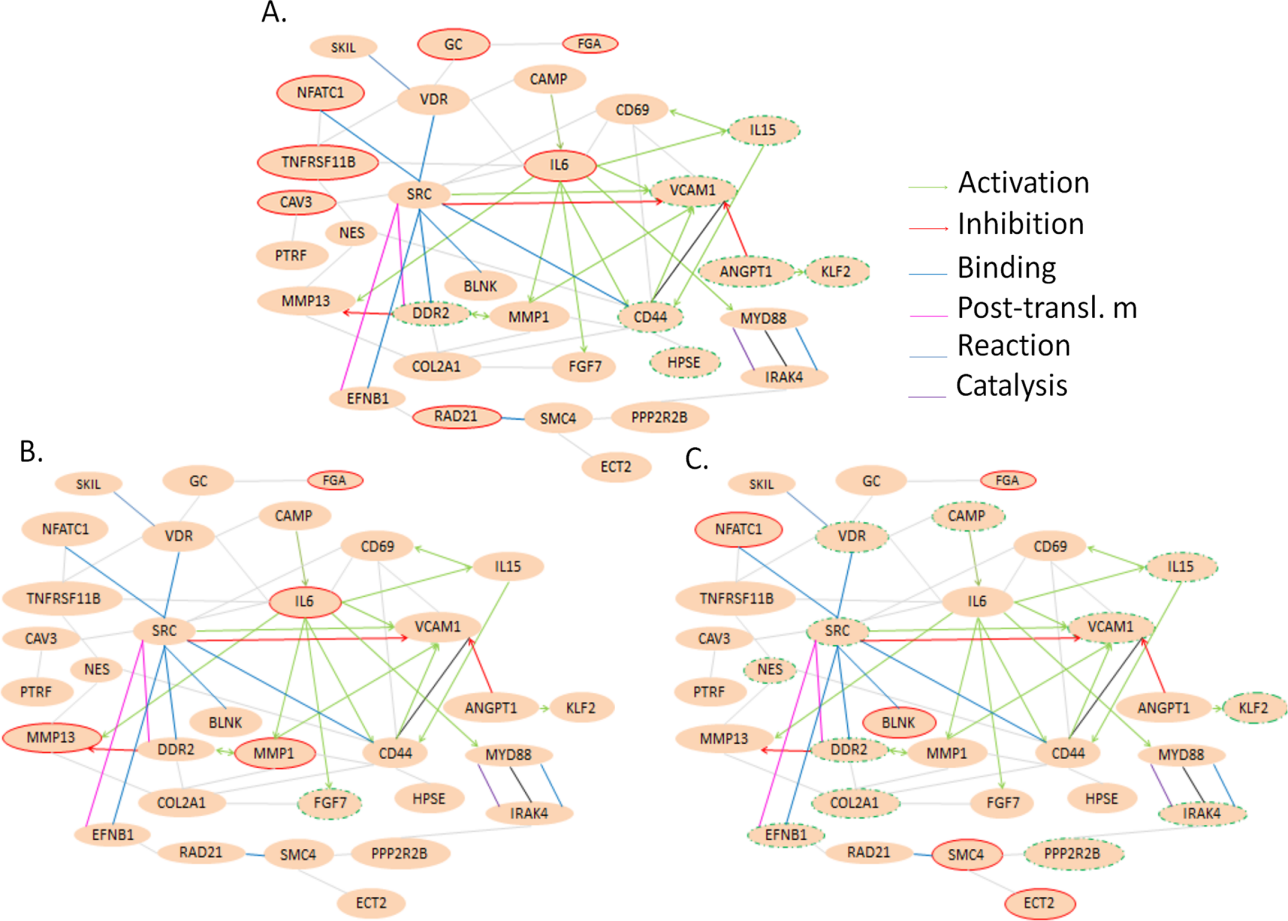
Network analysis of the differentially expressed genes in small yellow follicles of heat-stressed broiler-type B strain Taiwan country chickens. (A) H2R0, recovered for 0 h after heat stress; (B) H2R2, recovered for 2 h after heat stress; (C) H2R6, recovered for 6 h after heat stress.

The functional annotation pathway analysis of the differentially expressed genes and their interrelationships are depicted in Fig 4. These networks were associated with the biological functions of reproduction, responses to stress, and regulation of such responses. The major upregulated genes in the network after heat stress and recovery for 0 h were *IL6*, *GC*, *FGA*, *NFACT1*, *TNFRSF11B*, *CAV3*, and *RAD21*; for 2 h were *IL6*, *FGA*, *MMP1*, and *MMP13*; and for 6 h were *FGA*, *NFACT1*, *BLNK*, *SMC4*, and *ECT2* (S1 Table). The major downregulated genes in the network after heat stress and recovery for 0 h were *CD44*, *IL15*, *DDR2*, *KLF2*, *VCAM1*, and *ANGPT1*; for 2 h was *FGF7* only; and for 6 h were *SRC*, *VDR*, *NES*, *DDR2*, *IL1*5, *CAMP*, *KLF2*, *VCAM1*, *EFNB1*, *IRAK4*, *COL2A1*, and *PPP2R2B* (S2 Table).

### Validation of representative differentially expressed genes in the SYFs of broiler-type B strain TCCs after acute heat stress

Through functional annotation pathway analysis, 8 significantly changed genes revealed through microarray analysis were further validated using qRT-PCR (Table 2). The coefficient of variation of Cp value of GAPDH in the 4 groups ranged from 1.2% to 2.3% and implied that the heat stress did not affect its expression. Consistent with the microarray analysis, *HSP25*, *IL6*, *VTG2*, and *MMP13* were upregulated after heat stress. *COL2A1* and *KLF2* expressions were reduced by the acute heat stress in both the microarray and qRT-PCR analyses. *PTRF* and *DDR2* expression of qRT-PCR differed from those of the microarray analysis, and *DDR2* was upregulated after 2-h recovery in the qRT-PCR analysis. *PTRF* expression did not significantly differ after acute heat treatment in the qRT-PCR analysis.

**Table 2:** Multiples of changes of significantly differentially expressed genes in small yellow follicles of broiler-type B strain Taiwan country chickens after acute heat stress determined using microarray and quantitative reverse transcription polymerase chain reaction analyses.

Fold change*	Gene^a^							
	*HSP25*	*IL6*	*VTG2*	*MMP13*	*PTRF*	*COL2A1*	*DDR2*	*KLF2*
H2R0/CTL								
M	34.42	2.54	8.88	0.76	2.18	0.68	0.41	0.32
Q	54.40	1.93	3.37	0.96	0.91	0.81	1.09	0.27
H2R2/CTL								
M	38.20	2.11	1.21	3.21	2.08	0.98	0.82	0.95
Q	36.39	3.05	1.01	3.40	0.84	0.48	3.98	0.32
H2R6/CTL								
M	10.46	0.96	1.75	0.73	2.03	0.43	0.41	0.43
Q	8.40	1.82	1.65	0.82	0.74	0.53	0.94	0.34

* Multiples of changes of two-fold or higher increase or decrease were defined as different (p < 0.05). The fold expression or repression of the target gene were normalized using glyceraldehyde-3-phosphate dehydrogenase as an internal control gene.

^a^ Abbreviations: *HSP25*, heat shock protein 25; *IL6*, interleukin 6; *VTG2*, vitellogenin 2; *MMP13*, metalloproteinase 13; *PTRF*, polymerase I and transcript release factor; *COL2A1*, type II alpha 1 collagen; *DDR2*, discoidin domain receptor tyrosine kinase 2; *KLF2*, Kruppel-like factor 2.

## Discussions

### Effect of acute heat stress on physiological parameters and gene expressions in the SYFs of broiler-type B strain TCCs

Numerous studies have shown that heat stress affects egg production, egg weight, egg quality, and shell quality in chickens [22,23,24,25]. Few studies, however, have explored the global changes of gene expressions in the ovarian follicles. The results of the current study showed that the respiratory rate and body temperatures of heat-stressed hens increased significantly during acute heat stress and normalized after recovery at 25°C, which is consistent with the a previous report on roosters [13]. Global gene expression changes in ovarian SYFs were associated with metabolic, developmental, immune system, multicellular organismal, apoptotic, and cellular processes, apoptosis, biological regulation, localization, response to stimulus, biological adhesion, cellular component organization (biogenesis), and reproduction changes in the SYFs after acute heat stress (Fig 3).

### Heat shock protein family genes and other stress response related genes were induced in response to acute heat stress in the SYFs of broiler-type B strain TCCs

*HSP25* expression was significantly upregulated after acute heat stress (S1 Table; Table 2). *HSP25* is a small heat shock protein (sHSP) belonging to a family of conserved and ubiquitously expressed proteins [26]. *HSP25* stabilizes the unfolding proteins and prevents them from precipitating in cells [27]. Moreover, *HSP25* refolds numerous unfolding proteins and cooperates with other chaperones when organisms are recovered under optimal environmental conditions [28,29]. The elevated *HSP25* expression in this study suggests that *HSP25* facilitates protein refolding and chaperoning for preventing protein denaturation through acute heat insults in SYFs.

Acute phase response (APR) is a systemic and cellular reaction provoked by local or systemic disturbances in homeostasis caused by pathogen infection, tissue injury, trauma, stress, surgery, neoplasia, and immune disorders [30,31]. Numerous responses, including the production of proinflammatory cytokines (e.g., *IL6*, *IL1β*, and *TNF-α*) have been reported [32,33]. Furthermore, APR maintains physical homeostasis by activating the innate immune responses. *IL6* production during APR suppresses the production of proinflammatory cytokines without hampering the other anti-inflammatory cytokines [34]. *IL6* expression was significantly increased in the SYF (S1 Table; Table 2). Functional annotation analysis suggested that *IL6* upregulates interleukin 15 (*IL15*), matrix metalloproteinase-1 (*MMP-1*), matrix metalloproteinase-13 (*MMP-13*), fibroblast growth factor 7 (*FGF7*), vascular cell adhesion molecule 1 (*VCAM-1*), myeloid differentiation primary response 88 (*MYD88*), and *CD44* (Fig 4). However, the expression of *FGF7* and *VCAM-1* was downregulated, suggesting that epithelial cell injuries were exacerbated by acute stress [35,36]. Xing et al. [37] demonstrated that *IL6* is critical in controlling the extent of local and systemic acute inflammatory responses, particularly the levels of proinflammatory cytokines. Because functional pathway analysis showed that the differentially expressed genes were primarily associated with the biological processes of reproduction, response to stress, and regulation of these responses (Fig 3), *IL6* may initiate a protective mechanism against damage induced by heat stress in the SYF cells.

*KLF2*, a eukaryotic zinc finger transcription factor, has been reported to regulate various gene expressions in response to shear stress of vasculature endothelial cells for establishing and maintaining endothelial function [38,39]. *KLF2* has 3 carboxy-terminal zinc fingers with high homology to *KLF4*, the expression of which was significantly upregulated after heat stress in several tissues [40]. Liu et al. [40] reported that the overexpression of *KLF4* increased the mortality of C2C12 murine myogenic cells. Conversely, *KLF4* deficiency reduced C2C12 cell injury after heat stress [40]. *KLF2* expression was significantly downregulated (S2 Table; Table 2), implying that *KFL2* play a role in preventing SYF damage in hens exposed to acute heat stress.

### Acute heat stress may cause damage to the SYFs of broiler-type B strain TCCs

In chickens, vitellogenin, the major precursor protein of yolk, is synthesized in the liver [41]. Three vitellogenin genes exist, and the *VTG2* transcript is the most abundant [42]. *VTG2* expression in SYF was significantly increased after acute heat stress (Table 2). The role of upregulated *VTG2* expression in response to acute heat stress in chickens SYFs remains unknown. In this study, the expression of *MMP1* was upregulated after acute heat stress (Table 2). MMPs are zinc-dependent endopeptidases capable of degrading various extracellular matrix components [43,44]. Furthermore, MMPs play a critical role in follicular extracellular remodeling in mammalian ovaries [45]. Park et al. [46] reported that heat shock increased the *MMP1* and *MMP3* expression through an autocrine interleukin-6 loop. *IL6* inhibition by a monoclonal antibody significantly reduced the *MMP1* and *MMP13* expression in response to heat shock. *MMP1* expression was stimulated by a follicle-stimulating hormone, luteinizing hormone, progesterone, and estrogen, and remained low in the preovulatory follicles but increased in postovulatory follicles in chicken ovaries [45]. *MMP1* upregulation after heat stress thus may be disturbed by disordered secretion of sex hormones and can induce matrix disintegration in the follicles. This suggestion was further confirmed by the *COL2A1* downregulation and the transient upregulation of *MMP13* because of heat stress. *DDR2* induces *MMP13* expression [47], and *COL2A1* plays a critical role in collagen synthesis [48] and shares a majority of the total collagen genes in the ovary [49]. Liang et al. [49] reported that large amounts of misfolded procollagen were synthesized and retained in the dilated endoplasmic reticulum in *COL2A1* knockout mice [48]. In addition, *COL2A1* downregulation was observed in hypothyroid ovarian tissue, accompanied by the upregulation of *MMP1*, *MMP8*, and *MMP13* [49]. Thus, the downregulation of *COL2A1* and upregulation of *MMP1*, *MMP13*, and *IL-6* after acute heat stress suggest the proteolytic disintegration of the structural matrix and inflamed damage of the follicle cells after acute heat insults. In this study, *DDR2* was downregulated in H2R0 and H2R6 in the microarray analysis after acute heat stress.

*PTRF*, also known as cavin-1, participated in the dissociation of transcription complexes [50,51]. *PTRF* was recently reported to respond to mechanical stress by disassembling caveolae, [52] which, as a compact and rigid microdomain on the plasma membranes, has been implicated in several biological processes, including cell signaling, lipid regulation, and endocytosis [53]. Mechanical stress, such as osmotic swelling and unsymmetrical stretching, results in the rapid disappearance of caveolae [54]. The inner surface of caveolae is coated with a scaffolding protein formed by caveolin members [53]. *CAV3* concentration is significantly increased in damaged chicken muscle [55]. *PTRF* expression was significantly upregulated after acute stress, and *CAV3* expression was significantly upregulated at 0 h of recovery after heat stress (S2 Table; Table 2). These results indicate membrane permeability damaged by acute heat stress in SYF cells.

## Conclusions

Heat stress affects SYF gene expression in broiler-type B strain TCCs. The differentially expressed genes participated in such biological processes as metabolic, cellular, and developmental processes and biological regulation. Functional pathway analysis showed that *IL6* is a key regulator in the networks and connects the processes of reproduction, responses to stress, and regulation of such responses. The upregulation of heat shock protein 25, interleukin 6, metallopeptidase 1, and metalloproteinase 13, and downregulation of type II alpha 1 collagen, discoidin domain receptor tyrosine kinase 2, and Kruppel-like factor 2 suggest that acute heat stress induces proteolytic disintegration of the structural matrix and inflamed damage and adaptive responses of follicle cell gene expressions.

## Supporting Information

S1 Table Upregulated genes in the small yellow follicle of hens of B strain TCCs after acute heat stress. (DOCX)

S2 Table Downregulated genes in the small yellow follicle of hens of B strain TCCs after acute heat stress. (DOCX)
