# Supplementary material for: Annotation of Differential Gene Expression in Small Yellow Follicles of a Broiler-Type Strain of Taiwan Country Chickens in Response to Acute Heat Stress
Source: PLoS One. 2015 Nov 20;10(11):e0143418. doi: 10.1371/journal.pone.0143418 (PMC4654548; doi:10.1371/journal.pone.0143418)
Supplement: S1 Table — (DOCX) [file pone.0143418.s001.docx]

**S1 Table. Upregulated genes in the small yellow follicle of hens of B strain TCCs after acute heat stress**

| Probe name | Fold change | | | Gene symbol | UniGene ID | Description |
| --- | --- | --- | --- | --- | --- | --- |
|  | H2R0/CTL | H2R2/CTL | H2R6/CTL |  |  |  |
| Metabolic process | | | | | | |
| A_87_P106168 | 1.35 | -1.06 | 2.05 | AASS |  | PREDICTED: Gallus gallus aminoadipate-semialdehyde synthase (AASS), mRNA [XM_416001] |
| A_87_P036630 | -1.23 | 2.02 | -1.07 | ABCC5 | Gga.5635 | Gallus gallus mRNA for hypothetical protein, clone 10p3 [AJ720118] |
| A_87_P036478 | -1.29 | 2.01 | 1.07 | ASNS | Gga.22299 | Gallus gallus asparagine synthetase (glutamine-hydrolyzing) (ASNS), mRNA [NM_001030977] |
| A_87_P015251 | 1.29 | 1.19 | 2.06 | ATP11A | Gga.50119 | Gallus gallus finished cDNA, clone ChEST875n2 [CR388768] |
| A_87_P006367 | 1.29 | -1.06 | 2.03 | BIRC6 |  | PREDICTED: Gallus gallus baculoviral IAP repeat containing 6 (BIRC6), mRNA [XM_419512] |
| A_87_P082031 | 1.14 | -1.44 | 2.13 | CASP18 | Gga.32077 | Gallus gallus initiator caspase (CASP18), mRNA [NM_001044689] |
| A_87_P115053 | 2.90 | -1.21 | -1.12 | CAV3 | Gga.9266 | Gallus gallus finished cDNA, clone ChEST356f5 [BX932348] |
| A_87_P032473 | 2.04 | 1.01 | 1.60 | CDC42BPA | Gga.35105 | Gallus gallus CDC42 binding protein kinase alpha (DMPK-like) (CDC42BPA), mRNA [NM_001199443] |
| A_87_P035687 | 2.21 | -1.10 | -1.84 | CELF1 | Gga.14943 | Gallus gallus CUGBP, Elav-like family member 1 (CELF1), mRNA [NM_001012521] |
| A_87_P035181 | -1.05 | 2.07 | 1.18 | DCLRE1B | Gga.12786 | Gallus gallus DNA cross-link repair 1B (DCLRE1B), mRNA [NM_001031500] |
| A_87_P035324 | 1.31 | 1.18 | 2.18 | DLAT |  | PREDICTED: Gallus gallus dihydrolipoamide S-acetyltransferase (DLAT), mRNA [XM_417933] |
| A_87_P116043 | 2.16 | 1.02 | 1.41 | DLX1 | Gga.10508 | Gallus gallus distal-less homeobox 1 (DLX1), mRNA [NM_001045842] |
| A_87_P294568 | 1.87 | -1.04 | 2.15 | DNAJC13 | Gga.23616 | PREDICTED: Gallus gallus DnaJ (Hsp40) homolog, subfamily C, member 13 (DNAJC13), transcript variant X4, mRNA [XM_418787] |
| A_87_P035539 | 1.34 | -1.06 | 2.12 | DOCK10 | Gga.54975 | Gallus gallus mRNA for hypothetical protein, clone 33h8 [AJ851792] |
| A_87_P031832 | 1.03 | 1.05 | 2.05 | DYNC2H1 | Gga.26348 | 603592843F1 CSEQCHN34 Gallus gallus cDNA clone ChEST559e12 5', mRNA sequence [BU242831] |
| A_87_P013562 | 1.98 | 1.23 | 2.46 | ECT2 | Gga.51179 | Gallus gallus finished cDNA, clone ChEST261n20 [CR391299] |
| A_87_P016073 | 1.30 | -1.10 | 2.04 |  | Gga.55190 | Gallus gallus finished cDNA, clone ChEST436j12 [CR387046] |
| A_87_P081591 | -1.37 | 2.98 | -1.21 |  | Gga.53240 | gPGC_EST00523 Embryonic gonadal PGC cDNA Library Gallus gallus cDNA 5', mRNA sequence [DR410681] |
| A_87_P131118 | 2.31 | 1.21 | 1.91 |  | Gga.3772 | Gallus gallus T, brachyury homolog (mouse) (T), mRNA [ENSGALT00000018726] |
| A_87_P009759 | 2.08 | -1.10 | 2.28 |  |  | Gallus gallus MDP62 mRNA for muscle-derived protein 62, complete cds [D89964] |
| A_87_P024145 | 1.52 | -1.03 | 2.08 |  | Gga.55070 | Gallus gallus finished cDNA, clone ChEST307a5 [BX930940] |
| A_87_P015978 | 2.18 | 1.25 | -1.30 |  | Gga.50379 | Gallus gallus finished cDNA, clone ChEST775i6 [CR387204] |
| A_87_P100596 | 3.53 | 1.21 | -1.06 | GAL9 | Gga.4562 | Gallus gallus Gal 9 (GAL9), mRNA [NM_001001611] |
| A_87_P250503 | 2.16 | -1.45 | 1.38 | HHEX | Gga.3316 | Gallus gallus hematopoietically expressed homeobox (HHEX), mRNA [NM_205252] |
| A_87_P103668 | 2.44 | 1.40 | 1.91 | IRX2 |  | IRX2 iroquois homeobox 2 [ Gallus gallus (chicken) ] [ENSGALT00000021554] |
| A_87_P101136 | 1.72 | 1.23 | 2.35 | LARP4B |  | LARP4B La ribonucleoprotein domain family, member 4B [ Gallus gallus (chicken) ] [ENSGALT00000010791] |
| A_87_P009671 | 3.41 | -1.51 | 3.26 | LGSN | Gga.33717 | Gallus gallus lengsin, lens protein with glutamine synthetase domain (LGSN), mRNA [NM_001038586] |
| A_87_P014772 | 1.55 | 1.23 | 2.92 | LRRCC1 | Gga.17564 | Gallus gallus finished cDNA, clone ChEST303j2 [CR389519] |
| A_87_P092081 | 1.76 | 1.03 | 2.25 | MAST4 |  | PREDICTED: Gallus gallus microtubule associated serine/threonine kinase family member 4 (MAST4), transcript variant X12, mRNA [XM_424757] |
| A_87_P307473 | 2.51 | 1.06 | -1.57 | MID1 | Gga.159 | Gallus gallus midline 1 (Opitz/BBB syndrome) (MID1), mRNA [NM_204129] |
| A_87_P111518 | -1.22 | 2.36 | 1.06 | MMP1 |  | PREDICTED: Gallus gallus matrix metallopeptidase 1 (interstitial collagenase) (MMP1), mRNA [XM_417176] |
| A_87_P037942 | -1.32 | 3.21 | -1.38 | MMP13 | Gga.398 | Gallus gallus matrix metalloproteinase-13 (MMP-13) mRNA, partial cds [AF070478] |
| A_87_P123728 | -1.64 | 2.09 | -1.03 | MMP16 | Gga.2098 | Gallus gallus finished cDNA, clone ChEST952g10 [CR389196] |
| A_87_P243798 | 2.10 | 1.05 | -1.13 | MMS19 | Gga.17499 | Gallus gallus finished cDNA, clone ChEST1014o14 [CR390000] |
| A_87_P104513 | 1.69 | 1.22 | 2.12 | MYBL1 | Gga.14885 | Gallus gallus v-myb myeloblastosis viral oncogene homolog (avian)-like 1 (MYBL1), mRNA [NM_205232] |
| A_87_P024166 | 1.45 | -1.07 | 2.24 | MYCBP2 | Gga.50473 | Gallus gallus finished cDNA, clone ChEST966c16 [BX930845] |
| A_87_P151338 | -1.06 | 1.63 | 2.03 | NETO2 | Gga.10834 | 603130374F1 CSEQCHL14 Gallus gallus cDNA clone ChEST106c19 5', mRNA sequence [BU113756] |
| A_87_P169748 | 2.35 | -1.04 | 2.70 | NFATC1 |  | PREDICTED: Gallus gallus nuclear factor of activated T-cells, cytoplasmic, calcineurin-dependent 1 (NFATC1), transcript variant X4, mRNA [XM_418906] |
| A_87_P012630 | -1.33 | 2.59 | -1.04 | NPM3 | Gga.34672 | Gallus gallus finished cDNA, clone ChEST770n16 [CR406657] |
| A_87_P080746 | 1.43 | 1.41 | 2.15 | PCGF5 | Gga.14951 | Gallus gallus finished cDNA, clone ChEST234m6 [CR386353] |
| A_87_P093126 | 1.66 | 1.66 | 2.28 | PCSK1 |  | PREDICTED: Gallus gallus proprotein convertase subtilisin/kexin type 1 (PCSK1), mRNA [XM_003643060] |
| A_87_P036525 | 1.06 | 2.00 | 1.15 | PDIA4 | Gga.2205 | Gallus gallus protein disulfide isomerase family A, member 4 (PDIA4), mRNA [NM_001006370] |
| A_87_P104813 | 1.65 | -1.32 | 2.38 | PDP1 | Gga.23731 | Gallus gallus pyruvate dehyrogenase phosphatase catalytic subunit 1 (PDP1), nuclear gene encoding mitochondrial protein, mRNA [NM_001199910] |
| A_87_P117523 | 1.05 | 2.32 | -1.02 | PSAT1 | Gga.1510 | Gallus gallus finished cDNA, clone ChEST128f23. [BX935484] |
| A_87_P009772 | 2.18 | 2.08 | 2.03 | PTRF | Gga.977 | Gallus gallus mRNA for leucine-zipper protein (a variant of D26315), complete cds, clone CLFEST58-29 [D82080] |
| A_87_P036893 | 2.28 | 1.12 | -1.54 | RAD21 | Gga.4474 | Gallus gallus RAD21 homolog (S. pombe) (RAD21), mRNA [NM_001030950] |
| A_87_P082371 | 1.92 | 1.07 | 2.53 | RAPGEF4 |  | PREDICTED: Gallus gallus Rap guanine nucleotide exchange factor (GEF) 4 (RAPGEF4), transcript variant X4, mRNA [XM_426579] |
| A_87_P130978 | 2.30 | 1.01 | -1.66 | RARB | Gga.2668 | Gallus gallus retinoic acid receptor, beta (RARB), mRNA [NM_205326] |
| A_87_P102861 | 2.14 | 1.17 | 1.84 | RNF144B |  | PREDICTED: Gallus gallus ring finger protein 144B (RNF144B), transcript variant X5, mRNA [XM_418918] |
| A_87_P036112 | 1.18 | 2.35 | -1.20 | SDF2L1 | Gga.21846 | Gallus gallus mRNA for hypothetical protein, clone 28i23 [AJ720874] |
| A_87_P077881 | 1.62 | 1.23 | 2.35 | SKIL | Gga.42224 | Gallus gallus finished cDNA, clone ChEST660i10 [CR523941] |
| A_87_P097331 | 1.02 | 2.19 | 1.10 | SLC1A4 |  | PREDICTED: Gallus gallus solute carrier family 1 (glutamate/neutral amino acid transporter), member 4 (SLC1A4), mRNA [XM_001232899] |
| A_87_P035179 | 1.17 | 2.72 | 2.03 | SLC34A2 | Gga.216 | Gallus gallus solute carrier family 34 (sodium phosphate), member 2 (SLC34A2), mRNA [NM_204474] |
| A_87_P284808 | 1.73 | -1.21 | 2.21 | SMC4 | Gga.29302 | Gallus gallus structural maintenance of chromosomes 4 (SMC4), mRNA [NM_204518] |
| A_87_P058056 | 2.07 | 1.18 | -1.39 | SNTN | Gga.50530 | Gallus gallus sentan, cilia apical structure protein (SNTN), mRNA [NM_001142847] |
| A_87_P061276 | 1.14 | 2.32 | -1.88 | STC1 |  | PREDICTED: Gallus gallus stanniocalcin 1 (STC1), transcript variant X2, mRNA [XM_425760] |
| A_87_P096551 | 2.25 | -1.09 | -1.25 | STK32B | Gga.11676 | PREDICTED: Gallus gallus serine/threonine kinase 32B (STK32B), transcript variant X2, mRNA [XM_420796] |
| A_87_P018998 | 2.18 | -1.22 | -1.85 | TRIM42 | Gga.30227 | TRIM42 tripartite motif containing 42 [ Gallus gallus (chicken) ] [ENSGALT00000008477] |
| A_87_P035000 | 1.60 | -1.03 | 2.14 | USP32 | Gga.8855 | PREDICTED: Gallus gallus ubiquitin specific peptidase 32 (USP32), transcript variant X3, mRNA [XM_415892] |
| A_87_P099051 | 1.34 | 2.26 | 1.46 | VNN1 | Gga.13152 | Gallus gallus vanin 1 (VNN1), mRNA [NM_001039288] |
| A_87_P010627 | 1.20 | 1.11 | 2.06 | ZFYVE20 | Gga.49731 | gonad_EST02924 Embryonic gonad cDNA Library Gallus gallus cDNA 5', mRNA sequence [CV855448] |
| A_87_P092286 | 1.93 | -1.47 | 2.57 | ZNF366 |  | PREDICTED: Gallus gallus zinc finger protein 366 (ZNF366), mRNA [XM_429153] |
| A_87_P038200 | 1.67 | 1.22 | 2.35 | BLNK | Gga.3738 | Gallus gallus B-cell linker (BLNK), mRNA [NM_204908] |
| A_87_P115053 | 2.90 | -1.21 | -1.12 | CAV3 | Gga.9266 | Gallus gallus finished cDNA, clone ChEST356f5 [BX932348] |
| A_87_P032473 | 2.04 | 1.01 | 1.60 | CDC42BPA | Gga.35105 | Gallus gallus CDC42 binding protein kinase alpha (DMPK-like) (CDC42BPA), mRNA [NM_001199443] |
| A_87_P035687 | 2.21 | -1.10 | -1.84 | CELF1 | Gga.14943 | Gallus gallus CUGBP, Elav-like family member 1 (CELF1), mRNA [NM_001012521] |
| A_87_P066706 | 2.79 | -1.05 | 3.72 | CLTC | Gga.2120 | Gallus gallus clathrin, heavy chain (Hc) (CLTC), mRNA [NM_001080117] |
| A_87_P095216 | -1.14 | -1.20 | 3.00 | COL25A1 |  | PREDICTED: Gallus gallus collagen, type XXV, alpha 1 (COL25A1), transcript variant X2, mRNA [XM_427455] |
| A_87_P110843 | 1.36 | -1.20 | 2.12 | DIAPH3 |  | PREDICTED: Gallus gallus diaphanous homolog 3 (Drosophila) (DIAPH3), transcript variant X2, mRNA [XM_417020] |
| A_87_P294568 | 1.87 | -1.04 | 2.15 | DNAJC13 | Gga.23616 | PREDICTED: Gallus gallus DnaJ (Hsp40) homolog, subfamily C, member 13 (DNAJC13), transcript variant X4, mRNA [XM_418787] |
| A_87_P035539 | 1.34 | -1.06 | 2.12 | DOCK10 | Gga.54975 | Gallus gallus mRNA for hypothetical protein, clone 33h8 [AJ851792] |
| A_87_P031832 | 1.03 | 1.05 | 2.05 | DYNC2H1 | Gga.26348 | 603592843F1 CSEQCHN34 Gallus gallus cDNA clone ChEST559e12 5', mRNA sequence [BU242831] |
| A_87_P016073 | 1.30 | -1.10 | 2.04 |  | Gga.55190 | Gallus gallus finished cDNA, clone ChEST436j12 [CR387046] |
| A_87_P009759 | 2.08 | -1.10 | 2.28 |  |  | Gallus gallus MDP62 mRNA for muscle-derived protein 62, complete cds [D89964] |
| A_87_P014620 | 2.07 | -1.30 | 2.02 |  | Gga.54185 | Gallus gallus finished cDNA, clone ChEST317o19 [CR389742] |
| A_87_P015978 | 2.18 | 1.25 | -1.30 |  | Gga.50379 | Gallus gallus finished cDNA, clone ChEST775i6 [CR387204] |
| A_87_P009826 | -1.25 | 2.09 | -1.30 | EPYC | Gga.3583 | Gallus gallus epiphycan (EPYC), mRNA [NM_001004382] |
| A_87_P117178 | 2.46 | 1.18 | -1.04 | FGA | Gga.34280 | Gallus gallus fibrinogen alpha chain (FGA), transcript variant 2, mRNA [NM_205356] |
| A_87_P037453 | 2.54 | 2.11 | -1.04 | IL6 | Gga.2769 | Gallus gallus interleukin 6 (interferon, beta 2) (IL6), mRNA [NM_204628] |
| A_87_P112143 | 2.32 | 1.09 | -1.04 | LOC769834 |  | LOC769834 trophoblast glycoprotein-like [ Gallus gallus (chicken) ] [ENSGALT00000036350] |
| A_87_P014670 | 1.19 | -1.20 | 2.15 | LRP2 | Gga.54772 | Gallus gallus finished cDNA, clone ChEST437i20 [CR389664] |
| A_87_P014772 | 1.55 | 1.23 | 2.92 | LRRCC1 | Gga.17564 | Gallus gallus finished cDNA, clone ChEST303j2 [CR389519] |
| A_87_P229828 | 1.70 | 1.14 | 2.16 | MAP1B |  | MAP1B microtubule-associated protein 1B [ Gallus gallus (chicken) ] [ENSGALT00000024188] |
| A_87_P092081 | 1.76 | 1.03 | 2.25 | MAST4 |  | PREDICTED: Gallus gallus microtubule associated serine/threonine kinase family member 4 (MAST4), transcript variant X12, mRNA [XM_424757] |
| A_87_P104513 | 1.69 | 1.22 | 2.12 | MYBL1 | Gga.14885 | Gallus gallus v-myb myeloblastosis viral oncogene homolog (avian)-like 1 (MYBL1), mRNA [NM_205232] |
| A_87_P151338 | -1.06 | 1.63 | 2.03 | NETO2 | Gga.10834 | 603130374F1 CSEQCHL14 Gallus gallus cDNA clone ChEST106c19 5', mRNA sequence [BU113756] |
| A_87_P104813 | 1.65 | -1.32 | 2.38 | PDP1 | Gga.23731 | Gallus gallus pyruvate dehyrogenase phosphatase catalytic subunit 1 (PDP1), nuclear gene encoding mitochondrial protein, mRNA [NM_001199910] |
| A_87_P036893 | 2.28 | 1.12 | -1.54 | RAD21 | Gga.4474 | Gallus gallus RAD21 homolog (S. pombe) (RAD21), mRNA [NM_001030950] |
| A_87_P082371 | 1.92 | 1.07 | 2.53 | RAPGEF4 |  | PREDICTED: Gallus gallus Rap guanine nucleotide exchange factor (GEF) 4 (RAPGEF4), transcript variant X4, mRNA [XM_426579] |
| A_87_P130978 | 2.30 | 1.01 | -1.66 | RARB | Gga.2668 | Gallus gallus retinoic acid receptor, beta (RARB), mRNA [NM_205326] |
| A_87_P077881 | 1.62 | 1.23 | 2.35 | SKIL | Gga.42224 | Gallus gallus finished cDNA, clone ChEST660i10 [CR523941] |
| A_87_P097331 | 1.02 | 2.19 | 1.10 | SLC1A4 |  | PREDICTED: Gallus gallus solute carrier family 1 (glutamate/neutral amino acid transporter), member 4 (SLC1A4), mRNA [XM_001232899] |
| A_87_P284808 | 1.73 | -1.21 | 2.21 | SMC4 | Gga.29302 | Gallus gallus structural maintenance of chromosomes 4 (SMC4), mRNA [NM_204518] |
| A_87_P058056 | 2.07 | 1.18 | -1.39 | SNTN | Gga.50530 | Gallus gallus sentan, cilia apical structure protein (SNTN), mRNA [NM_001142847] |
| A_87_P077211 | 2.07 | 1.01 | -1.29 | SPSB4 | Gga.29010 | PREDICTED: Gallus gallus splA [XM_426685] |
| A_87_P017732 | 2.70 | 1.14 | 2.48 | SSX2IP | Gga.11681 | Gallus gallus synovial sarcoma, X breakpoint 2 interacting protein (SSX2IP), mRNA [NM_001044656] |
| A_87_P061276 | 1.14 | 2.32 | -1.88 | STC1 |  | PREDICTED: Gallus gallus stanniocalcin 1 (STC1), transcript variant X2, mRNA [XM_425760] |
| A_87_P096551 | 2.25 | -1.09 | -1.25 | STK32B | Gga.11676 | PREDICTED: Gallus gallus serine/threonine kinase 32B (STK32B), transcript variant X2, mRNA [XM_420796] |
| A_87_P092211 | 1.75 | 2.77 | -1.77 | SV2C |  | PREDICTED: Gallus gallus synaptic vesicle glycoprotein 2C (SV2C), transcript variant X2, mRNA [XM_429151] |
| A_87_P009679 | -1.83 | 2.26 | -1.08 | TLR15 | Gga.26502 | Gallus gallus toll-like receptor 15 (TLR15), mRNA [NM_001037835] |
| A_87_P018558 | 2.03 | 1.60 | 1.35 | TNFRSF11B | Gga.1812 | Gallus gallus finished cDNA, clone ChEST175o17 [CR352482] |
| A_87_P099051 | 1.34 | 2.26 | 1.46 | VNN1 | Gga.13152 | Gallus gallus vanin 1 (VNN1), mRNA [NM_001039288] |
| A_87_P015251 | 1.29 | 1.19 | 2.06 | ATP11A | Gga.50119 | Gallus gallus finished cDNA, clone ChEST875n2 [CR388768] |
| A_87_P006367 | 1.29 | -1.06 | 2.03 | BIRC6 |  | PREDICTED: Gallus gallus baculoviral IAP repeat containing 6 (BIRC6), mRNA [XM_419512] |
| A_87_P082031 | 1.14 | -1.44 | 2.13 | CASP18 | Gga.32077 | Gallus gallus initiator caspase (CASP18), mRNA [NM_001044689] |
| A_87_P115053 | 2.90 | -1.21 | -1.12 | CAV3 | Gga.9266 | Gallus gallus finished cDNA, clone ChEST356f5 [BX932348] |
| A_87_P095216 | -1.14 | -1.20 | 3.00 | COL25A1 |  | PREDICTED: Gallus gallus collagen, type XXV, alpha 1 (COL25A1), transcript variant X2, mRNA [XM_427455] |
| A_87_P116043 | 2.16 | 1.02 | 1.41 | DLX1 | Gga.10508 | Gallus gallus distal-less homeobox 1 (DLX1), mRNA [NM_001045842] |
| A_87_P035539 | 1.34 | -1.06 | 2.12 | DOCK10 | Gga.54975 | Gallus gallus mRNA for hypothetical protein, clone 33h8 [AJ851792] |
| A_87_P016073 | 1.30 | -1.10 | 2.04 |  | Gga.55190 | Gallus gallus finished cDNA, clone ChEST436j12 [CR387046] |
| A_87_P131118 | 2.31 | 1.21 | 1.91 |  | Gga.3772 | Gallus gallus T, brachyury homolog (mouse) (T), mRNA [ENSGALT00000018726] |
| A_87_P024145 | 1.52 | -1.03 | 2.08 |  | Gga.55070 | Gallus gallus finished cDNA, clone ChEST307a5 [BX930940] |
| A_87_P250503 | 2.16 | -1.45 | 1.38 | HHEX | Gga.3316 | Gallus gallus hematopoietically expressed homeobox (HHEX), mRNA [NM_205252] |
| A_87_P037453 | 2.54 | 2.11 | -1.04 | IL6 | Gga.2769 | Gallus gallus interleukin 6 (interferon, beta 2) (IL6), mRNA [NM_204628] |
| A_87_P103668 | 2.44 | 1.40 | 1.91 | IRX2 |  | IRX2 iroquois homeobox 2 [ Gallus gallus (chicken) ] [ENSGALT00000021554] |
| A_87_P014670 | 1.19 | -1.20 | 2.15 | LRP2 | Gga.54772 | Gallus gallus finished cDNA, clone ChEST437i20 [CR389664] |
| A_87_P014772 | 1.55 | 1.23 | 2.92 | LRRCC1 | Gga.17564 | Gallus gallus finished cDNA, clone ChEST303j2 [CR389519] |
| A_87_P243798 | 2.10 | 1.05 | -1.13 | MMS19 | Gga.17499 | Gallus gallus finished cDNA, clone ChEST1014o14 [CR390000] |
| A_87_P104513 | 1.69 | 1.22 | 2.12 | MYBL1 | Gga.14885 | Gallus gallus v-myb myeloblastosis viral oncogene homolog (avian)-like 1 (MYBL1), mRNA [NM_205232] |
| A_87_P151338 | -1.06 | 1.63 | 2.03 | NETO2 | Gga.10834 | 603130374F1 CSEQCHL14 Gallus gallus cDNA clone ChEST106c19 5', mRNA sequence [BU113756] |
| A_87_P169748 | 2.35 | -1.04 | 2.70 | NFATC1 |  | PREDICTED: Gallus gallus nuclear factor of activated T-cells, cytoplasmic, calcineurin-dependent 1 (NFATC1), transcript variant X4, mRNA [XM_418906] |
| A_87_P080746 | 1.43 | 1.41 | 2.15 | PCGF5 | Gga.14951 | Gallus gallus finished cDNA, clone ChEST234m6 [CR386353] |
| A_87_P104813 | 1.65 | -1.32 | 2.38 | PDP1 | Gga.23731 | Gallus gallus pyruvate dehyrogenase phosphatase catalytic subunit 1 (PDP1), nuclear gene encoding mitochondrial protein, mRNA [NM_001199910] |
| A_87_P082371 | 1.92 | 1.07 | 2.53 | RAPGEF4 |  | PREDICTED: Gallus gallus Rap guanine nucleotide exchange factor (GEF) 4 (RAPGEF4), transcript variant X4, mRNA [XM_426579] |
| A_87_P130978 | 2.30 | 1.01 | -1.66 | RARB | Gga.2668 | Gallus gallus retinoic acid receptor, beta (RARB), mRNA [NM_205326] |
| A_87_P077881 | 1.62 | 1.23 | 2.35 | SKIL | Gga.42224 | Gallus gallus finished cDNA, clone ChEST660i10 [CR523941] |
| A_87_P061276 | 1.14 | 2.32 | -1.88 | STC1 |  | PREDICTED: Gallus gallus stanniocalcin 1 (STC1), transcript variant X2, mRNA [XM_425760] |
| A_87_P018558 | 2.03 | 1.60 | 1.35 | TNFRSF11B | Gga.1812 | Gallus gallus finished cDNA, clone ChEST175o17 [CR352482] |
| A_87_P092286 | 1.93 | -1.47 | 2.57 | ZNF366 |  | PREDICTED: Gallus gallus zinc finger protein 366 (ZNF366), mRNA [XM_429153] |
| A_87_P082031 | 1.14 | -1.44 | 2.13 | CASP18 | Gga.32077 | Gallus gallus initiator caspase (CASP18), mRNA [NM_001044689] |
| A_87_P032473 | 2.04 | 1.01 | 1.60 | CDC42BPA | Gga.35105 | Gallus gallus CDC42 binding protein kinase alpha (DMPK-like) (CDC42BPA), mRNA [NM_001199443] |
| A_87_P035687 | 2.21 | -1.10 | -1.84 | CELF1 | Gga.14943 | Gallus gallus CUGBP, Elav-like family member 1 (CELF1), mRNA [NM_001012521] |
| A_87_P095216 | -1.14 | -1.20 | 3.00 | COL25A1 |  | PREDICTED: Gallus gallus collagen, type XXV, alpha 1 (COL25A1), transcript variant X2, mRNA [XM_427455] |
| A_87_P116043 | 2.16 | 1.02 | 1.41 | DLX1 | Gga.10508 | Gallus gallus distal-less homeobox 1 (DLX1), mRNA [NM_001045842] |
| A_87_P016073 | 1.30 | -1.10 | 2.04 |  | Gga.55190 | Gallus gallus finished cDNA, clone ChEST436j12 [CR387046] |
| A_87_P131118 | 2.31 | 1.21 | 1.91 |  | Gga.3772 | Gallus gallus T, brachyury homolog (mouse) (T), mRNA [ENSGALT00000018726] |
| A_87_P014620 | 2.07 | -1.30 | 2.02 |  | Gga.54185 | Gallus gallus finished cDNA, clone ChEST317o19 [CR389742] |
| A_87_P037453 | 2.54 | 2.11 | -1.04 | IL6 | Gga.2769 | Gallus gallus interleukin 6 (interferon, beta 2) (IL6), mRNA [NM_204628] |
| A_87_P103668 | 2.44 | 1.40 | 1.91 | IRX2 |  | IRX2 iroquois homeobox 2 [ Gallus gallus (chicken) ] [ENSGALT00000021554] |
| A_87_P014670 | 1.19 | -1.20 | 2.15 | LRP2 | Gga.54772 | Gallus gallus finished cDNA, clone ChEST437i20 [CR389664] |
| A_87_P104513 | 1.69 | 1.22 | 2.12 | MYBL1 | Gga.14885 | Gallus gallus v-myb myeloblastosis viral oncogene homolog (avian)-like 1 (MYBL1), mRNA [NM_205232] |
| A_87_P151338 | -1.06 | 1.63 | 2.03 | NETO2 | Gga.10834 | 603130374F1 CSEQCHL14 Gallus gallus cDNA clone ChEST106c19 5', mRNA sequence [BU113756] |
| A_87_P077881 | 1.62 | 1.23 | 2.35 | SKIL | Gga.42224 | Gallus gallus finished cDNA, clone ChEST660i10 [CR523941] |
| A_87_P009679 | -1.83 | 2.26 | -1.08 | TLR15 | Gga.26502 | Gallus gallus toll-like receptor 15 (TLR15), mRNA [NM_001037835] |
| A_87_P018558 | 2.03 | 1.60 | 1.35 | TNFRSF11B | Gga.1812 | Gallus gallus finished cDNA, clone ChEST175o17 [CR352482] |
| Immune system process | | | | | | |
| A_87_P036630 | -1.23 | 2.02 | -1.07 | ABCC5 | Gga.5635 | Gallus gallus mRNA for hypothetical protein, clone 10p3 [AJ720118] |
| A_87_P095216 | -1.14 | -1.20 | 3.00 | COL25A1 |  | PREDICTED: Gallus gallus collagen, type XXV, alpha 1 (COL25A1), transcript variant X2, mRNA [XM_427455] |
| A_87_P016073 | 1.30 | -1.10 | 2.04 |  | Gga.55190 | Gallus gallus finished cDNA, clone ChEST436j12 [CR387046] |
| A_87_P014620 | 2.07 | -1.30 | 2.02 |  | Gga.54185 | Gallus gallus finished cDNA, clone ChEST317o19 [CR389742] |
| A_87_P015978 | 2.18 | 1.25 | -1.30 |  | Gga.50379 | Gallus gallus finished cDNA, clone ChEST775i6 [CR387204] |
| A_87_P100596 | 3.53 | 1.21 | -1.06 | GAL9 | Gga.4562 | Gallus gallus Gal 9 (GAL9), mRNA [NM_001001611] |
| A_87_P037453 | 2.54 | 2.11 | -1.04 | IL6 | Gga.2769 | Gallus gallus interleukin 6 (interferon, beta 2) (IL6), mRNA [NM_204628] |
| A_87_P151338 | -1.06 | 1.63 | 2.03 | NETO2 | Gga.10834 | 603130374F1 CSEQCHL14 Gallus gallus cDNA clone ChEST106c19 5', mRNA sequence [BU113756] |
| A_87_P058056 | 2.07 | 1.18 | -1.39 | SNTN | Gga.50530 | Gallus gallus sentan, cilia apical structure protein (SNTN), mRNA [NM_001142847] |
| A_87_P018558 | 2.03 | 1.60 | 1.35 | TNFRSF11B | Gga.1812 | Gallus gallus finished cDNA, clone ChEST175o17 [CR352482] |
| Localization | | | | | | |
| A_87_P036630 | -1.23 | 2.02 | -1.07 | ABCC5 | Gga.5635 | Gallus gallus mRNA for hypothetical protein, clone 10p3 [AJ720118] |
| A_87_P015251 | 1.29 | 1.19 | 2.06 | ATP11A | Gga.50119 | Gallus gallus finished cDNA, clone ChEST875n2 [CR388768] |
| A_87_P115053 | 2.90 | -1.21 | -1.12 | CAV3 | Gga.9266 | Gallus gallus finished cDNA, clone ChEST356f5 [BX932348] |
| A_87_P066706 | 2.79 | -1.05 | 3.72 | CLTC | Gga.2120 | Gallus gallus clathrin, heavy chain (Hc) (CLTC), mRNA [NM_001080117] |
| A_87_P095216 | -1.14 | -1.20 | 3.00 | COL25A1 |  | PREDICTED: Gallus gallus collagen, type XXV, alpha 1 (COL25A1), transcript variant X2, mRNA [XM_427455] |
| A_87_P035539 | 1.34 | -1.06 | 2.12 | DOCK10 | Gga.54975 | Gallus gallus mRNA for hypothetical protein, clone 33h8 [AJ851792] |
| A_87_P031832 | 1.03 | 1.05 | 2.05 | DYNC2H1 | Gga.26348 | 603592843F1 CSEQCHN34 Gallus gallus cDNA clone ChEST559e12 5', mRNA sequence [BU242831] |
| A_87_P016073 | 1.30 | -1.10 | 2.04 |  | Gga.55190 | Gallus gallus finished cDNA, clone ChEST436j12 [CR387046] |
| A_87_P014620 | 2.07 | -1.30 | 2.02 |  | Gga.54185 | Gallus gallus finished cDNA, clone ChEST317o19 [CR389742] |
| A_87_P111818 | 1.59 | 1.03 | 2.36 |  | Gga.27550 | Gallus gallus finished cDNA, clone ChEST16o3 [ENSGALT00000025550] |
| A_87_P015978 | 2.18 | 1.25 | -1.30 |  | Gga.50379 | Gallus gallus finished cDNA, clone ChEST775i6 [CR387204] |
| A_87_P158058 | 1.77 | 3.16 | 1.77 | KCNK12 |  | PREDICTED: Gallus gallus potassium channel, subfamily K, member 12 (KCNK12), partial mRNA [XM_003640904] |
| A_87_P204283 | 2.20 | 1.24 | -1.56 | NAPB | Gga.9615 | Gallus gallus N-ethylmaleimide-sensitive factor attachment protein, beta (NAPB), mRNA [NM_001199430] |
| A_87_P151338 | -1.06 | 1.63 | 2.03 | NETO2 | Gga.10834 | 603130374F1 CSEQCHL14 Gallus gallus cDNA clone ChEST106c19 5', mRNA sequence [BU113756] |
| A_87_P322607 | 2.47 | 1.01 | -2.04 | SLC12A9 |  | PREDICTED: Gallus gallus solute carrier family 12 (potassium/chloride transporters), member 9 (SLC12A9), partial mRNA [XM_003641791] |
| A_87_P097331 | 1.02 | 2.19 | 1.10 | SLC1A4 |  | PREDICTED: Gallus gallus solute carrier family 1 (glutamate/neutral amino acid transporter), member 4 (SLC1A4), mRNA [XM_001232899] |
| A_87_P035179 | 1.17 | 2.72 | 2.03 | SLC34A2 | Gga.216 | Gallus gallus solute carrier family 34 (sodium phosphate), member 2 (SLC34A2), mRNA [NM_204474] |
| Response to stimulus | | | | | | |
| A_87_P036630 | -1.23 | 2.02 | -1.07 | ABCC5 | Gga.5635 | Gallus gallus mRNA for hypothetical protein, clone 10p3 [AJ720118] |
| A_87_P095216 | -1.14 | -1.20 | 3.00 | COL25A1 |  | PREDICTED: Gallus gallus collagen, type XXV, alpha 1 (COL25A1), transcript variant X2, mRNA [XM_427455] |
| A_87_P016073 | 1.30 | -1.10 | 2.04 |  | Gga.55190 | Gallus gallus finished cDNA, clone ChEST436j12 [CR387046] |
| A_87_P014620 | 2.07 | -1.30 | 2.02 |  | Gga.54185 | Gallus gallus finished cDNA, clone ChEST317o19 [CR389742] |
| A_87_P015978 | 2.18 | 1.25 | -1.30 |  | Gga.50379 | Gallus gallus finished cDNA, clone ChEST775i6 [CR387204] |
| A_87_P100596 | 3.53 | 1.21 | -1.06 | GAL9 | Gga.4562 | Gallus gallus Gal 9 (GAL9), mRNA [NM_001001611] |
| A_87_P014670 | 1.19 | -1.20 | 2.15 | LRP2 | Gga.54772 | Gallus gallus finished cDNA, clone ChEST437i20 [CR389664] |
| A_87_P151338 | -1.06 | 1.63 | 2.03 | NETO2 | Gga.10834 | 603130374F1 CSEQCHL14 Gallus gallus cDNA clone ChEST106c19 5', mRNA sequence [BU113756] |
| A_87_P169748 | 2.35 | -1.04 | 2.70 | NFATC1 |  | PREDICTED: Gallus gallus nuclear factor of activated T-cells, cytoplasmic, calcineurin-dependent 1 (NFATC1), transcript variant X4, mRNA [XM_418906] |
| A_87_P077881 | 1.62 | 1.23 | 2.35 | SKIL | Gga.42224 | Gallus gallus finished cDNA, clone ChEST660i10 [CR523941] |
| A_87_P018558 | 2.03 | 1.60 | 1.35 | TNFRSF11B | Gga.1812 | Gallus gallus finished cDNA, clone ChEST175o17 [CR352482] |
| Multicellular organismal process | | | | | | |
| A_87_P035687 | 2.21 | -1.10 | -1.84 | CELF1 | Gga.14943 | Gallus gallus CUGBP, Elav-like family member 1 (CELF1), mRNA [NM_001012521] |
| A_87_P066706 | 2.79 | -1.05 | 3.72 | CLTC | Gga.2120 | Gallus gallus clathrin, heavy chain (Hc) (CLTC), mRNA [NM_001080117] |
| A_87_P095216 | -1.14 | -1.20 | 3.00 | COL25A1 |  | PREDICTED: Gallus gallus collagen, type XXV, alpha 1 (COL25A1), transcript variant X2, mRNA [XM_427455] |
| A_87_P116043 | 2.16 | 1.02 | 1.41 | DLX1 | Gga.10508 | Gallus gallus distal-less homeobox 1 (DLX1), mRNA [NM_001045842] |
| A_87_P016073 | 1.30 | -1.10 | 2.04 |  | Gga.55190 | Gallus gallus finished cDNA, clone ChEST436j12 [CR387046] |
| A_87_P014620 | 2.07 | -1.30 | 2.02 |  | Gga.54185 | Gallus gallus finished cDNA, clone ChEST317o19 [CR389742] |
| A_87_P009826 | -1.25 | 2.09 | -1.30 | EPYC | Gga.3583 | Gallus gallus epiphycan (EPYC), mRNA [NM_001004382] |
| A_87_P158058 | 1.77 | 3.16 | 1.77 | KCNK12 |  | PREDICTED: Gallus gallus potassium channel, subfamily K, member 12 (KCNK12), partial mRNA [XM_003640904] |
| A_87_P014670 | 1.19 | -1.20 | 2.15 | LRP2 | Gga.54772 | Gallus gallus finished cDNA, clone ChEST437i20 [CR389664] |
| A_87_P151338 | -1.06 | 1.63 | 2.03 | NETO2 | Gga.10834 | 603130374F1 CSEQCHL14 Gallus gallus cDNA clone ChEST106c19 5', mRNA sequence [BU113756] |
| A_87_P097331 | 1.02 | 2.19 | 1.10 | SLC1A4 |  | PREDICTED: Gallus gallus solute carrier family 1 (glutamate/neutral amino acid transporter), member 4 (SLC1A4), mRNA [XM_001232899] |
| A_87_P092211 | 1.75 | 2.77 | -1.77 | SV2C |  | PREDICTED: Gallus gallus synaptic vesicle glycoprotein 2C (SV2C), transcript variant X2, mRNA [XM_429151] |
| Biological adhesion | | | | | | |
| A_87_P032473 | 2.04 | 1.01 | 1.60 | CDC42BPA | Gga.35105 | Gallus gallus CDC42 binding protein kinase alpha (DMPK-like) (CDC42BPA), mRNA [NM_001199443] |
| A_87_P095216 | -1.14 | -1.20 | 3.00 | COL25A1 |  | PREDICTED: Gallus gallus collagen, type XXV, alpha 1 (COL25A1), transcript variant X2, mRNA [XM_427455] |
| A_87_P015978 | 2.18 | 1.25 | -1.30 |  | Gga.50379 | Gallus gallus finished cDNA, clone ChEST775i6 [CR387204] |
| A_87_P009826 | -1.25 | 2.09 | -1.30 | EPYC | Gga.3583 | Gallus gallus epiphycan (EPYC), mRNA [NM_001004382] |
| A_87_P117178 | 2.46 | 1.18 | -1.04 | FGA | Gga.34280 | Gallus gallus fibrinogen alpha chain (FGA), transcript variant 2, mRNA [NM_205356] |
| A_87_P151338 | -1.06 | 1.63 | 2.03 | NETO2 | Gga.10834 | 603130374F1 CSEQCHL14 Gallus gallus cDNA clone ChEST106c19 5', mRNA sequence [BU113756] |
| A_87_P009679 | -1.83 | 2.26 | -1.08 | TLR15 | Gga.26502 | Gallus gallus toll-like receptor 15 (TLR15), mRNA [NM_001037835] |
| A_87_P099051 | 1.34 | 2.26 | 1.46 | VNN1 | Gga.13152 | Gallus gallus vanin 1 (VNN1), mRNA [NM_001039288] |
| Apoptotic process | | |  |  |  |  |
| A_87_P082031 | 1.14 | -1.44 | 2.13 | CASP18 | Gga.32077 | Gallus gallus initiator caspase (CASP18), mRNA [NM_001044689] |
| A_87_P016073 | 1.30 | -1.10 | 2.04 |  | Gga.55190 | Gallus gallus finished cDNA, clone ChEST436j12 [CR387046] |
| A_87_P037453 | 2.54 | 2.11 | -1.04 | IL6 | Gga.2769 | Gallus gallus interleukin 6 (interferon, beta 2) (IL6), mRNA [NM_204628] |
| A_87_P104513 | 1.69 | 1.22 | 2.12 | MYBL1 | Gga.14885 | Gallus gallus v-myb myeloblastosis viral oncogene homolog (avian)-like 1 (MYBL1), mRNA [NM_205232] |
| A_87_P077881 | 1.62 | 1.23 | 2.35 | SKIL | Gga.42224 | Gallus gallus finished cDNA, clone ChEST660i10 [CR523941] |
| A_87_P018558 | 2.03 | 1.60 | 1.35 | TNFRSF11B | Gga.1812 | Gallus gallus finished cDNA, clone ChEST175o17 [CR352482] |
| Cellular component organization or biogenesis | | | | | | |
| A_87_P095216 | -1.14 | -1.20 | 3.00 | COL25A1 |  | PREDICTED: Gallus gallus collagen, type XXV, alpha 1 (COL25A1), transcript variant X2, mRNA [XM_427455] |
| A_87_P284808 | 1.73 | -1.21 | 2.21 | SMC4 | Gga.29302 | Gallus gallus structural maintenance of chromosomes 4 (SMC4), mRNA [NM_204518] |
| A_87_P031832 | 1.03 | 1.05 | 2.05 | DYNC2H1 | Gga.26348 | 603592843F1 CSEQCHN34 Gallus gallus cDNA clone ChEST559e12 5', mRNA sequence [BU242831] |
| A_87_P032473 | 2.04 | 1.01 | 1.60 | CDC42BPA | Gga.35105 | Gallus gallus CDC42 binding protein kinase alpha (DMPK-like) (CDC42BPA), mRNA [NM_001199443] |
| reproduction |  |  |  |  |  |  |
| A_87_P014620 | 2.07 | -1.30 | 2.02 |  | Gga.54185 | Gallus gallus finished cDNA, clone ChEST317o19 [CR389742] |
| A_87_P016073 | 1.30 | -1.10 | 2.04 |  | Gga.55190 | Gallus gallus finished cDNA, clone ChEST436j12 [CR387046] |
