# Supplementary material for: Annotation of Differential Gene Expression in Small Yellow Follicles of a Broiler-Type Strain of Taiwan Country Chickens in Response to Acute Heat Stress
Source: PLoS One. 2015 Nov 20;10(11):e0143418. doi: 10.1371/journal.pone.0143418 (PMC4654548; doi:10.1371/journal.pone.0143418)
Supplement: S2 Table — (DOCX) [file pone.0143418.s002.docx]

**S2 Table. Downregulated genes in the small yellow follicle of hens of B strain TCCs after acute heat stress**

| Probe name | Fold change | | | Gene symbol | UniGene ID | Description |
| --- | --- | --- | --- | --- | --- | --- |
|  | H2R0/CTL | H2R2/CTL | H2R6/CTL |  |  |  |
| Metabolic process | | | | |  |  |
| A_87_P289883 | -3.25 | -2.11 | -3.09 | ABI3 |  | PREDICTED: Gallus gallus ABI family, member 3 (ABI3), transcript variant X12, mRNA [XM_418110] |
| A_87_P022330 | -1.48 | -1.14 | -2.02 | AFMID | Gga.42222 | Gallus gallus finished cDNA, clone ChEST990l21 [BX934979] |
| A_87_P004170 | -1.33 | -2.48 | -1.35 | AGXT2L2 |  | PREDICTED: Gallus gallus alanine-glyoxylate aminotransferase 2-like 2 (AGXT2L2), transcript variant X4, mRNA [XM_414688] |
| A_87_P008907 | -1.55 | -1.60 | -2.21 | ALDH1A1 | Gga.4119 | Gallus gallus aldehyde dehydrogenase 1 family, member A1 (ALDH1A1), mRNA [NM_204577] |
| A_87_P225208 | 1.00 | -2.02 | -1.28 | APOBEC2 | Gga.12112 | Gallus gallus finished cDNA, clone ChEST164i18 [BX935898] |
| A_87_P037142 | -2.05 | -1.45 | -1.62 | ARHGAP15 | Gga.22226 | Gallus gallus Rho GTPase activating protein 15 (ARHGAP15), mRNA [NM_001008476] |
| A_87_P018539 | -2.16 | -1.18 | 1.19 | ARHGEF10 | Gga.55643 | Gallus gallus finished cDNA, clone ChEST824a5 [CR352520] |
| A_87_P282773 | -1.28 | -1.71 | -2.43 | ARIH1 | Gga.53210 | Gallus gallus ariadne homolog, ubiquitin-conjugating enzyme E2 binding protein, 1 (Drosophila) (ARIH1), mRNA [NM_001030387] |
| A_87_P096166 | -2.22 | 1.31 | -1.54 | ATP10D | Gga.9127 | gPGC_EST02444 Embryonic gonadal PGC cDNA Library Gallus gallus cDNA 5', mRNA sequence [DR412602] |
| A_87_P023657 | -2.00 | 1.22 | -2.20 | C1H21orf7 | Gga.10636 | Gallus gallus finished cDNA, clone ChEST156j9. [BX932098] |
| A_87_P066751 | -2.11 | -1.53 | -1.57 | CA4 | Gga.54539 | Gallus gallus finished cDNA, clone ChEST209p6 [BX931094] |
| A_87_P061961 | -2.20 | -1.31 | -1.74 | CAMK4 | Gga.16160 | Gallus gallus finished cDNA, clone ChEST892d10. [CR407245] |
| A_87_P192318 | -2.03 | -1.43 | -2.11 | CRYL1 | Gga.9304 | Gallus gallus crystallin, lambda 1 (CRYL1), mRNA [NM_001030830] |
| A_87_P095071 | -2.09 | -1.41 | -1.75 | CSGALNACT1 |  | CSGALNACT1 chondroitin sulfate N-acetylgalactosaminyltransferase 1 [ Gallus gallus (chicken) ] [ENSGALT00000016467] |
| A_87_P144763 | -1.65 | -1.47 | -2.05 | DNAJC12 | Gga.1230 | Gallus gallus DnaJ (Hsp40) homolog, subfamily C, member 12 (DNAJC12), mRNA [NM_001199530] |
| A_87_P073381 | -1.22 | -1.42 | -2.13 | DPYSL3 | Gga.9493 | Gallus gallus dihydropyrimidinase-like 3 (DPYSL3), mRNA [NM_204493] |
| A_87_P037894 | -1.68 | -2.63 | -3.69 | EEF1B2 | Gga.4763 | Gallus gallus eukaryotic translation elongation factor 1 beta 2 (EEF1B2), mRNA [NM_204901] |
| A_87_P065676 | -1.64 | -2.48 | -1.62 | FBLIM1 |  | PREDICTED: Gallus gallus filamin binding LIM protein 1 (FBLIM1), transcript variant X3, mRNA [XM_417617] |
| A_87_P129023 | -4.77 | -2.33 | -8.27 | GAL2 | Gga.495 | Gallus gallus gallinacin 2 (GAL2), transcript variant 2, mRNA [NM_001201399] |
| A_87_P035105 | -6.19 | -2.76 | -10.26 | GAL7 | Gga.5606 | Gallus gallus Gal 7 (GAL7), mRNA [NM_001001194] |
| A_87_P018823 | -2.02 | 1.64 | 1.75 | GCFC1 | Gga.14840 | Gallus gallus finished cDNA, clone ChEST972b23 [CR338813] |
| A_87_P024671 | -2.07 | 1.28 | -1.69 | HKDC1 | Gga.11459 | Gallus gallus finished cDNA, clone ChEST745a20 [BX929581] |
| A_87_P134638 | -2.18 | -1.67 | -1.56 | HPSE | Gga.950 | Gallus gallus heparanase (HPSE), mRNA [NM_204167] |
| A_87_P005649 | -1.91 | 1.25 | -2.51 | HSD17B2 |  | PREDICTED: Gallus gallus hydroxysteroid (17-beta) dehydrogenase 2 (HSD17B2), mRNA [XM_414168] |
| A_87_P135043 | -1.63 | -1.18 | -2.09 | IRAK4 |  | IRAK4 interleukin-1 receptor-associated kinase 4 [ Gallus gallus (chicken) ] [ENSGALT00000015616] |
| A_87_P062211 | -3.08 | -1.05 | -2.32 | KLF2 |  | PREDICTED: Gallus gallus Kruppel-like factor 2 (lung) (KLF2), mRNA [XM_418264] |
| A_87_P200203 | -2.88 | -1.04 | -3.16 | LAPTM5 |  | PREDICTED: Gallus gallus lysosomal protein transmembrane 5 (LAPTM5), transcript variant X4, mRNA [XM_417701] |
| A_87_P010892 | -2.80 | -1.74 | -2.96 | LOC428958 | Gga.21033 | Gallus gallus finished cDNA, clone ChEST786b9 [CR524445] |
| A_87_P072951 | -2.04 | -1.03 | -1.95 | MRPL22 | Gga.1889 | Gallus gallus finished cDNA, clone ChEST1016l14 [BX930154] |
| A_87_P008971 | -1.71 | -2.54 | -1.42 | MYH11 | Gga.3225 | Gallus gallus myosin, heavy chain 11, smooth muscle (MYH11), mRNA [NM_205274] |
| A_87_P102136 | -2.36 | -1.32 | -2.06 | NOD1 | Gga.40185 | Gallus gallus nucleotide-binding oligomerization domain-containing protein 1 (NOD1) mRNA, complete cds [JX465487] |
| A_87_P068481 | -3.69 | -1.04 | -2.46 | PPAPDC3 | Gga.31772 | 603586668F1 CSEQCHN72 Gallus gallus cDNA clone ChEST543h17 5-, mRNA sequence [BU365573] |
| A_87_P164148 | -1.42 | -1.15 | -2.02 | PPP2R2B |  | PPP2R2B protein phosphatase 2, regulatory subunit B, beta [ Gallus gallus (chicken) ] [ENSGALT00000012233] |
| A_87_P076551 | -2.21 | -1.29 | -2.36 | RASL12 | Gga.23848 | RASL12 RAS-like, family 12 [ Gallus gallus (chicken) ] [ENSGALT00000011886] |
| A_87_P215823 | -2.56 | -1.51 | -2.67 | RASSF2 | Gga.4577 | Gallus gallus Ras association (RalGDS/AF-6) domain family member 2 (RASSF2), mRNA [NM_001030884] |
| A_87_P078321 | 1.26 | -1.33 | -2.38 | RGS5 | Gga.54654 | Gallus gallus finished cDNA, clone ChEST202i3 [BX931079] |
| A_87_P036344 | -1.34 | 1.09 | -2.66 | RNF7 | Gga.6330 | Gallus gallus ring finger protein 7 (RNF7), mRNA [NM_001031307] |
| A_87_P106598 | -3.47 | -1.39 | -1.63 | RPS16 |  | RPS16 ribosomal protein S16 [ Gallus gallus (chicken) ] [ENSGALT00000016819] |
| A_87_P192808 | -2.12 | -1.29 | -1.20 | SEPT6 | Gga.21276 | Gallus gallus septin 6 (SEPT6), mRNA [NM_001031125] |
| A_87_P058471 | -2.73 | -1.92 | -5.65 | SERPINB10 | Gga.415 | Gallus gallus serpin peptidase inhibitor, clade B (ovalbumin), member 10 (SERPINB10), mRNA [NM_204897] |
| A_87_P078226 | -2.08 | -1.52 | -1.45 | SLC25A24 |  | PREDICTED: Gallus gallus solute carrier family 25 (mitochondrial carrier; phosphate carrier), member 24 (SLC25A24), mRNA [XM_422180] |
| A_87_P080476 | -2.21 | -1.63 | -1.69 | SORBS1 |  | PREDICTED: Gallus gallus sorbin and SH3 domain containing 1 (SORBS1), transcript variant X11, mRNA [XM_421625] |
| A_87_P130933 | 1.25 | -1.20 | -2.04 | SRC | Gga.46254 | c-src {5' region heterogeneity} [chickens, mRNA Partial, 273 nt] [S43587] |
| A_87_P056441 | 1.06 | -2.13 | -1.00 | SULT1B1 | Gga.39508 | Gallus gallus sulfotransferase family, cytosolic, 1B, member 1 (SULT1B1), mRNA [NM_204545] |
| A_87_P021532 | -4.86 | -1.04 | -3.12 | VCAM1 | Gga.10270 | Gallus gallus finished cDNA, clone ChEST607g6 [BX950651] |
| A_87_P013338 | -1.34 | -1.10 | -2.02 | VDR | Gga.584 | Gallus gallus vitamin D (1,25- dihydroxyvitamin D3) receptor (VDR), mRNA [NM_205098] |
| Cellular process | | | |  |  |  |
| A_87_P023657 | -2.00 | 1.22 | -2.20 | C1H21orf7 | Gga.10636 | Gallus gallus finished cDNA, clone ChEST156j9. [BX932098] |
| A_87_P289883 | -3.25 | -2.11 | -3.09 | ABI3 |  | PREDICTED: Gallus gallus ABI family, member 3 (ABI3), transcript variant X12, mRNA [XM_418110] |
| A_87_P150538 | -2.02 | 1.04 | -1.37 | ANGPT1 | Gga.9471 | Gallus gallus angiopoietin 1 (ANGPT1), mRNA [NM_001199447] |
| A_87_P037142 | -2.05 | -1.45 | -1.62 | ARHGAP15 | Gga.22226 | Gallus gallus Rho GTPase activating protein 15 (ARHGAP15), mRNA [NM_001008476] |
| A_87_P061961 | -2.20 | -1.31 | -1.74 | CAMK4 | Gga.16160 | Gallus gallus finished cDNA, clone ChEST892d10. [CR407245] |
| A_87_P223853 | -2.63 | -1.75 | -2.27 | CD3D | Gga.998 | Gallus gallus breed ISA CD3 glycoprotein precursor mRNA, complete cds [AY574975] |
| A_87_P058637 | -1.62 | -1.24 | -2.02 | CHIR-A2 | Gga.42223 | Gallus gallus immunoglobulin-like receptor CHIR-A2 (CHIR-A2), mRNA [NM_001146138] |
| A_87_P294113 | -1.47 | -1.02 | -2.32 | COL2A1 | Gga.2839 | Gallus gallus collagen, type II, alpha 1 (COL2A1), mRNA [NM_204426] |
| A_87_P080031 | -1.44 | -1.38 | -2.11 | CTNNA3 | Gga.19961 | Gallus gallus catenin (cadherin-associated protein), alpha 3 (CTNNA3), mRNA [NM_001199536] |
| A_87_P144763 | -1.65 | -1.47 | -2.05 | DNAJC12 | Gga.1230 | Gallus gallus DnaJ (Hsp40) homolog, subfamily C, member 12 (DNAJC12), mRNA [NM_001199530] |
| A_87_P087131 | -2.29 | -1.37 | -2.54 |  | Gga.56295 | Gallus gallus partial mRNA for immunoglobulin-like receptor CHIR2D-831 precursor (CHIR2D-831 gene), strain R11 [FM200332] |
| A_87_P023284 | -2.66 | 1.26 | -1.21 |  | Gga.10684 | Gallus gallus finished cDNA, clone ChEST996h15 [BX932899] |
| A_87_P088043 | -2.86 | -1.35 | -2.55 |  | Gga.55581 | PREDICTED: Gallus gallus immunoglobulin superfamily member 1-like (LOC100858475), miscRNA [XR_140392] |
| A_87_P129823 | -1.85 | -1.29 | -2.16 |  | Gga.29219 | Gallus gallus partial mRNA for immunoglobulin-like receptor CHIR2D-716 precursor (CHIR2D-716 gene), strain M11 [FM200253] |
| A_87_P086946 | -1.86 | -1.23 | -2.89 |  |  | Uncharacterized protein [ENSGALT00000036122] |
| A_87_P065676 | -1.64 | -2.48 | -1.62 | FBLIM1 |  | PREDICTED: Gallus gallus filamin binding LIM protein 1 (FBLIM1), transcript variant X3, mRNA [XM_417617] |
| A_87_P024790 | -1.02 | -3.15 | 1.01 | FGF7 | Gga.11317 | Gallus gallus fibroblast growth factor 7 (FGF7), mRNA [NM_001012525] |
| A_87_P094751 | 1.99 | -1.74 | -2.01 | GLRB |  | PREDICTED: Gallus gallus glycine receptor, beta (GLRB), transcript variant X2, mRNA [XM_420379] |
| A_87_P024671 | -2.07 | 1.28 | -1.69 | HKDC1 | Gga.11459 | Gallus gallus finished cDNA, clone ChEST745a20 [BX929581] |
| A_87_P037924 | -2.38 | 1.26 | -1.44 | IFNAR1 | Gga.50569 | Gallus gallus interferon (alpha, beta and omega) receptor 1 (IFNAR1), mRNA [NM_204859] |
| A_87_P037851 | -4.28 | -1.21 | -2.43 | IL15 | Gga.42186 | Gallus gallus interleukin 15 (IL15), mRNA [NM_204571] |
| A_87_P135043 | -1.63 | -1.18 | -2.09 | IRAK4 |  | IRAK4 interleukin-1 receptor-associated kinase 4 [ Gallus gallus (chicken) ] [ENSGALT00000015616] |
| A_87_P016597 | -4.89 | 1.89 | -2.98 | LRRTM3 | Gga.16173 | Gallus gallus finished cDNA, clone ChEST131b14 [CR386266] |
| A_87_P008971 | -1.71 | -2.54 | -1.42 | MYH11 | Gga.3225 | Gallus gallus myosin, heavy chain 11, smooth muscle (MYH11), mRNA [NM_205274] |
| A_87_P154173 | -2.61 | 1.06 | -1.52 | PDZRN3 |  | PREDICTED: Gallus gallus PDZ domain containing RING finger 3 (PDZRN3), transcript variant X2, mRNA [XM_414432] |
| A_87_P117848 | -1.32 | -2.53 | -2.18 | PNOC | Gga.10041 | Gallus gallus finished cDNA, clone ChEST19i24 [BX936050] |
| A_87_P068481 | -3.69 | -1.04 | -2.46 | PPAPDC3 | Gga.31772 | 603586668F1 CSEQCHN72 Gallus gallus cDNA clone ChEST543h17 5-, mRNA sequence [BU365573] |
| A_87_P076551 | -2.21 | -1.29 | -2.36 | RASL12 | Gga.23848 | RASL12 RAS-like, family 12 [ Gallus gallus (chicken) ] [ENSGALT00000011886] |
| A_87_P215823 | -2.56 | -1.51 | -2.67 | RASSF2 | Gga.4577 | Gallus gallus Ras association (RalGDS/AF-6) domain family member 2 (RASSF2), mRNA [NM_001030884] |
| A_87_P078321 | 1.26 | -1.33 | -2.38 | RGS5 | Gga.54654 | Gallus gallus finished cDNA, clone ChEST202i3 [BX931079] |
| A_87_P036344 | -1.34 | 1.09 | -2.66 | RNF7 | Gga.6330 | Gallus gallus ring finger protein 7 (RNF7), mRNA [NM_001031307] |
| A_87_P192808 | -2.12 | -1.29 | -1.20 | SEPT6 | Gga.21276 | Gallus gallus septin 6 (SEPT6), mRNA [NM_001031125] |
| A_87_P035216 | -2.16 | 1.67 | 1.11 | SLA | Gga.8508 | Gallus gallus Src-like-adaptor (SLA), mRNA [NM_204391] |
| A_87_P080476 | -2.21 | -1.63 | -1.69 | SORBS1 |  | PREDICTED: Gallus gallus sorbin and SH3 domain containing 1 (SORBS1), transcript variant X11, mRNA [XM_421625] |
| A_87_P130933 | 1.25 | -1.20 | -2.04 | SRC | Gga.46254 | c-src {5' region heterogeneity} [chickens, mRNA Partial, 273 nt] [S43587] |
| A_87_P038218 | -1.18 | -1.61 | -2.29 | TGFBI | Gga.2582 | Gallus gallus transforming growth factor, beta-induced, 68kDa (TGFBI), mRNA [NM_205036] |
| A_87_P021532 | -4.86 | -1.04 | -3.12 | VCAM1 | Gga.10270 | Gallus gallus finished cDNA, clone ChEST607g6 [BX950651] |
| A_87_P013338 | -1.34 | -1.10 | -2.02 | VDR | Gga.584 | Gallus gallus vitamin D (1,25- dihydroxyvitamin D3) receptor (VDR), mRNA [NM_205098] |
| Biological regulation | | | | | | |
| A_87_P289883 | -3.25 | -2.11 | -3.09 | ABI3 |  | PREDICTED: Gallus gallus ABI family, member 3 (ABI3), transcript variant X12, mRNA [XM_418110] |
| A_87_P037142 | -2.05 | -1.45 | -1.62 | ARHGAP15 | Gga.22226 | Gallus gallus Rho GTPase activating protein 15 (ARHGAP15), mRNA [NM_001008476] |
| A_87_P096166 | -2.22 | 1.31 | -1.54 | ATP10D | Gga.9127 | gPGC_EST02444 Embryonic gonadal PGC cDNA Library Gallus gallus cDNA 5', mRNA sequence [DR412602] |
| A_87_P294113 | -1.47 | -1.02 | -2.32 | COL2A1 | Gga.2839 | Gallus gallus collagen, type II, alpha 1 (COL2A1), mRNA [NM_204426] |
| A_87_P037894 | -1.68 | -2.63 | -3.69 | EEF1B2 | Gga.4763 | Gallus gallus eukaryotic translation elongation factor 1 beta 2 (EEF1B2), mRNA [NM_204901] |
| A_87_P065676 | -1.64 | -2.48 | -1.62 | FBLIM1 |  | PREDICTED: Gallus gallus filamin binding LIM protein 1 (FBLIM1), transcript variant X3, mRNA [XM_417617] |
| A_87_P018823 | -2.02 | 1.64 | 1.75 | GCFC1 | Gga.14840 | Gallus gallus finished cDNA, clone ChEST972b23 [CR338813] |
| A_87_P024671 | -2.07 | 1.28 | -1.69 | HKDC1 | Gga.11459 | Gallus gallus finished cDNA, clone ChEST745a20 [BX929581] |
| A_87_P037851 | -4.28 | -1.21 | -2.43 | IL15 | Gga.42186 | Gallus gallus interleukin 15 (IL15), mRNA [NM_204571] |
| A_87_P062211 | -3.08 | -1.05 | -2.32 | KLF2 |  | PREDICTED: Gallus gallus Kruppel-like factor 2 (lung) (KLF2), mRNA [XM_418264] |
| A_87_P008971 | -1.71 | -2.54 | -1.42 | MYH11 | Gga.3225 | Gallus gallus myosin, heavy chain 11, smooth muscle (MYH11), mRNA [NM_205274] |
| A_87_P215823 | -2.56 | -1.51 | -2.67 | RASSF2 | Gga.4577 | Gallus gallus Ras association (RalGDS/AF-6) domain family member 2 (RASSF2), mRNA [NM_001030884] |
| A_87_P078321 | 1.26 | -1.33 | -2.38 | RGS5 | Gga.54654 | Gallus gallus finished cDNA, clone ChEST202i3 [BX931079] |
| A_87_P058471 | -2.73 | -1.92 | -5.65 | SERPINB10 | Gga.415 | Gallus gallus serpin peptidase inhibitor, clade B (ovalbumin), member 10 (SERPINB10), mRNA [NM_204897] |
| A_87_P013338 | -1.34 | -1.10 | -2.02 | VDR | Gga.584 | Gallus gallus vitamin D (1,25- dihydroxyvitamin D3) receptor (VDR), mRNA [NM_205098] |
| Developmental process | | |  |  |  |  |
| A_87_P061266 | -3.68 | 1.65 | -1.85 | ADAM28 | Gga.19451 | Gallus gallus finished cDNA, clone ChEST1015b9 [419529] |
| A_87_P150538 | -2.02 | 1.04 | -1.37 | ANGPT1 | Gga.9471 | Gallus gallus angiopoietin 1 (ANGPT1), mRNA [NM_001199447] |
| A_87_P061961 | -2.20 | -1.31 | -1.74 | CAMK4 | Gga.16160 | Gallus gallus finished cDNA, clone ChEST892d10. [CR407245] |
| A_87_P294113 | -1.47 | -1.02 | -2.32 | COL2A1 | Gga.2839 | Gallus gallus collagen, type II, alpha 1 (COL2A1), mRNA [NM_204426] |
| A_87_P080031 | -1.44 | -1.38 | -2.11 | CTNNA3 | Gga.19961 | Gallus gallus catenin (cadherin-associated protein), alpha 3 (CTNNA3), mRNA [NM_001199536] |
| A_87_P065676 | -1.64 | -2.48 | -1.62 | FBLIM1 |  | PREDICTED: Gallus gallus filamin binding LIM protein 1 (FBLIM1), transcript variant X3, mRNA [XM_417617] |
| A_87_P024790 | -1.02 | -3.15 | 1.01 | FGF7 | Gga.11317 | Gallus gallus fibroblast growth factor 7 (FGF7), mRNA [NM_001012525] |
| A_87_P094751 | 1.99 | -1.74 | -2.01 | GLRB |  | PREDICTED: Gallus gallus glycine receptor, beta (GLRB), transcript variant X2, mRNA [XM_420379] |
| A_87_P037851 | -4.28 | -1.21 | -2.43 | IL15 | Gga.42186 | Gallus gallus interleukin 15 (IL15), mRNA [NM_204571] |
| A_87_P054261 | -3.57 | -1.18 | -1.63 | IL16 | Gga.1307 | Gallus gallus interleukin 16 (lymphocyte chemoattractant factor) (IL16), mRNA [NM_204352] |
| A_87_P062211 | -3.08 | -1.05 | -2.32 | KLF2 |  | PREDICTED: Gallus gallus Kruppel-like factor 2 (lung) (KLF2), mRNA [XM_418264] |
| A_87_P016597 | -4.89 | 1.89 | -2.98 | LRRTM3 | Gga.16173 | Gallus gallus finished cDNA, clone ChEST131b14 [CR386266] |
| A_87_P008971 | -1.71 | -2.54 | -1.42 | MYH11 | Gga.3225 | Gallus gallus myosin, heavy chain 11, smooth muscle (MYH11), mRNA [NM_205274] |
| A_87_P102136 | -2.36 | -1.32 | -2.06 | NOD1 | Gga.40185 | Gallus gallus nucleotide-binding oligomerization domain-containing protein 1 (NOD1) mRNA, complete cds [JX465487] |
| A_87_P068481 | -3.69 | -1.04 | -2.46 | PPAPDC3 | Gga.31772 | 603586668F1 CSEQCHN72 Gallus gallus cDNA clone ChEST543h17 5-, mRNA sequence [BU365573] |
| A_87_P215823 | -2.56 | -1.51 | -2.67 | RASSF2 | Gga.4577 | Gallus gallus Ras association (RalGDS/AF-6) domain family member 2 (RASSF2), mRNA [NM_001030884] |
| A_87_P035216 | -2.16 | 1.67 | 1.11 | SLA | Gga.8508 | Gallus gallus Src-like-adaptor (SLA), mRNA [NM_204391] |
| A_87_P080476 | -2.21 | -1.63 | -1.69 | SORBS1 |  | PREDICTED: Gallus gallus sorbin and SH3 domain containing 1 (SORBS1), transcript variant X11, mRNA [XM_421625] |
| A_87_P130933 | 1.25 | -1.20 | -2.04 | SRC | Gga.46254 | c-src {5' region heterogeneity} [chickens, mRNA Partial, 273 nt] [S43587] |
| A_87_P038218 | -1.18 | -1.61 | -2.29 | TGFBI | Gga.2582 | Gallus gallus transforming growth factor, beta-induced, 68kDa (TGFBI), mRNA [NM_205036] |
| A_87_P021532 | -4.86 | -1.04 | -3.12 | VCAM1 | Gga.10270 | Gallus gallus finished cDNA, clone ChEST607g6 [BX950651] |
| A_87_P013338 | -1.34 | -1.10 | -2.02 | VDR | Gga.584 | Gallus gallus vitamin D (1,25- dihydroxyvitamin D3) receptor (VDR), mRNA [NM_205098] |
| Immune system process | | |  |  |  |  |
| A_87_P164148 | -1.42 | -1.15 | -2.02 | PPP2R2B |  | PPP2R2B protein phosphatase 2, regulatory subunit B, beta [ Gallus gallus (chicken) ] [ENSGALT00000012233] |
| A_87_P294113 | -1.47 | -1.02 | -2.32 | COL2A1 | Gga.2839 | Gallus gallus collagen, type II, alpha 1 (COL2A1), mRNA [NM_204426] |
| A_87_P058637 | -1.62 | -1.24 | -2.02 | CHIR-A2 | Gga.42223 | Gallus gallus immunoglobulin-like receptor CHIR-A2 (CHIR-A2), mRNA [NM_001146138] |
| A_87_P135043 | -1.63 | -1.18 | -2.09 | IRAK4 |  | IRAK4 interleukin-1 receptor-associated kinase 4 [ Gallus gallus (chicken) ] [ENSGALT00000015616] |
| A_87_P129823 | -1.85 | -1.29 | -2.16 |  | Gga.29219 | Gallus gallus partial mRNA for immunoglobulin-like receptor CHIR2D-716 precursor (CHIR2D-716 gene), strain M11 [FM200253] |
| A_87_P086946 | -1.86 | -1.23 | -2.89 |  |  | Uncharacterized protein [ENSGALT00000036122] |
| A_87_P035216 | -2.16 | 1.67 | 1.11 | SLA | Gga.8508 | Gallus gallus Src-like-adaptor (SLA), mRNA [NM_204391] |
| A_87_P061961 | -2.20 | -1.31 | -1.74 | CAMK4 | Gga.16160 | Gallus gallus finished cDNA, clone ChEST892d10. [CR407245] |
| A_87_P087131 | -2.29 | -1.37 | -2.54 |  | Gga.56295 | Gallus gallus partial mRNA for immunoglobulin-like receptor CHIR2D-831 precursor (CHIR2D-831 gene), strain R11 [FM200332] |
| A_87_P135053 | -2.34 | -1.05 | -2.03 | GLIPR1 | Gga.6848 | Gallus gallus GLI pathogenesis-related 1 (GLIPR1), mRNA [NM_001030743] |
| A_87_P102136 | -2.36 | -1.32 | -2.06 | NOD1 | Gga.40185 | Gallus gallus nucleotide-binding oligomerization domain-containing protein 1 (NOD1) mRNA, complete cds [JX465487] |
| A_87_P037924 | -2.38 | 1.26 | -1.44 | IFNAR1 | Gga.50569 | Gallus gallus interferon (alpha, beta and omega) receptor 1 (IFNAR1), mRNA [NM_204859] |
| A_87_P223853 | -2.63 | -1.75 | -2.27 | CD3D | Gga.998 | Gallus gallus breed ISA CD3 glycoprotein precursor mRNA, complete cds [AY574975] |
| A_87_P088043 | -2.86 | -1.35 | -2.55 |  | Gga.55581 | PREDICTED: Gallus gallus immunoglobulin superfamily member 1-like (LOC100858475), miscRNA [XR_140392] |
| A_87_P062211 | -3.08 | -1.05 | -2.32 | KLF2 |  | PREDICTED: Gallus gallus Kruppel-like factor 2 (lung) (KLF2), mRNA [XM_418264] |
| A_87_P054261 | -3.57 | -1.18 | -1.63 | IL16 | Gga.1307 | Gallus gallus interleukin 16 (lymphocyte chemoattractant factor) (IL16), mRNA [NM_204352] |
| A_87_P037851 | -4.28 | -1.21 | -2.43 | IL15 | Gga.42186 | Gallus gallus interleukin 15 (IL15), mRNA [NM_204571] |
| A_87_P129023 | -4.77 | -2.33 | -8.27 | GAL2 | Gga.495 | Gallus gallus gallinacin 2 (GAL2), transcript variant 2, mRNA [NM_001201399] |
| A_87_P021532 | -4.86 | -1.04 | -3.12 | VCAM1 | Gga.10270 | Gallus gallus finished cDNA, clone ChEST607g6 [BX950651] |
| A_87_P035105 | -6.19 | -2.76 | -10.26 | GAL7 | Gga.5606 | Gallus gallus Gal 7 (GAL7), mRNA [NM_001001194] |
| Localization | |  |  |  |  |  |
| A_87_P096166 | -2.22 | 1.31 | -1.54 | ATP10D | Gga.9127 | gPGC_EST02444 Embryonic gonadal PGC cDNA Library Gallus gallus cDNA 5', mRNA sequence [DR412602] |
| A_87_P294953 | -2.13 | -1.12 | -1.43 | CHMP2B | Gga.9706 | Gallus gallus chromatin modifying protein 2B (CHMP2B), mRNA [NM_001030792] |
| A_87_P294113 | -1.47 | -1.02 | -2.32 | COL2A1 | Gga.2839 | Gallus gallus collagen, type II, alpha 1 (COL2A1), mRNA [NM_204426] |
| A_87_P096041 | -2.09 | 1.36 | 1.07 | EXOC1 | Gga.22001 | EXOC1 exocyst complex component 1 [ Gallus gallus (chicken) ] [ENSGALT00000022376] |
| A_87_P094751 | 1.99 | -1.74 | -2.01 | GLRB |  | PREDICTED: Gallus gallus glycine receptor, beta (GLRB), transcript variant X2, mRNA [XM_420379] |
| A_87_P200203 | -2.88 | -1.04 | -3.16 | LAPTM5 |  | PREDICTED: Gallus gallus lysosomal protein transmembrane 5 (LAPTM5), transcript variant X4, mRNA [XM_417701] |
| A_87_P008971 | -1.71 | -2.54 | -1.42 | MYH11 | Gga.3225 | Gallus gallus myosin, heavy chain 11, smooth muscle (MYH11), mRNA [NM_205274] |
| A_87_P080266 | -2.66 | -1.42 | -1.77 | OIT3 | Gga.13291 | Gallus gallus finished cDNA, clone ChEST536i23 [CR354362] |
| A_87_P076551 | -2.21 | -1.29 | -2.36 | RASL12 | Gga.23848 | RASL12 RAS-like, family 12 [ Gallus gallus (chicken) ] [ENSGALT00000011886] |
| A_87_P078226 | -2.08 | -1.52 | -1.45 | SLC25A24 |  | PREDICTED: Gallus gallus solute carrier family 25 (mitochondrial carrier; phosphate carrier), member 24 (SLC25A24), mRNA [XM_422180] |
| A_87_P080476 | -2.21 | -1.63 | -1.69 | SORBS1 |  | PREDICTED: Gallus gallus sorbin and SH3 domain containing 1 (SORBS1), transcript variant X11, mRNA [XM_421625] |
| Response to stimulus | | | |  |  |  |
| A_87_P150538 | -2.02 | 1.04 | -1.37 | ANGPT1 | Gga.9471 | Gallus gallus angiopoietin 1 (ANGPT1), mRNA [NM_001199447] |
| A_87_P061961 | -2.20 | -1.31 | -1.74 | CAMK4 | Gga.16160 | Gallus gallus finished cDNA, clone ChEST892d10. [CR407245] |
| A_87_P223853 | -2.63 | -1.75 | -2.27 | CD3D | Gga.998 | Gallus gallus breed ISA CD3 glycoprotein precursor mRNA, complete cds [AY574975] |
| A_87_P058637 | -1.62 | -1.24 | -2.02 | CHIR-A2 | Gga.42223 | Gallus gallus immunoglobulin-like receptor CHIR-A2 (CHIR-A2), mRNA [NM_001146138] |
| A_87_P294113 | -1.47 | -1.02 | -2.32 | COL2A1 | Gga.2839 | Gallus gallus collagen, type II, alpha 1 (COL2A1), mRNA [NM_204426] |
| A_87_P087131 | -2.29 | -1.37 | -2.54 |  | Gga.56295 | Gallus gallus partial mRNA for immunoglobulin-like receptor CHIR2D-831 precursor (CHIR2D-831 gene), strain R11 [FM200332] |
| A_87_P088043 | -2.86 | -1.35 | -2.55 |  | Gga.55581 | PREDICTED: Gallus gallus immunoglobulin superfamily member 1-like (LOC100858475), miscRNA [XR_140392] |
| A_87_P129823 | -1.85 | -1.29 | -2.16 |  | Gga.29219 | Gallus gallus partial mRNA for immunoglobulin-like receptor CHIR2D-716 precursor (CHIR2D-716 gene), strain M11 [FM200253] |
| A_87_P086946 | -1.86 | -1.23 | -2.89 |  |  | Uncharacterized protein [ENSGALT00000036122] |
| A_87_P129023 | -4.77 | -2.33 | -8.27 | GAL2 | Gga.495 | Gallus gallus gallinacin 2 (GAL2), transcript variant 2, mRNA [NM_001201399] |
| A_87_P035105 | -6.19 | -2.76 | -10.26 | GAL7 | Gga.5606 | Gallus gallus Gal 7 (GAL7), mRNA [NM_001001194] |
| A_87_P037924 | -2.38 | 1.26 | -1.44 | IFNAR1 | Gga.50569 | Gallus gallus interferon (alpha, beta and omega) receptor 1 (IFNAR1), mRNA [NM_204859] |
| A_87_P054261 | -3.57 | -1.18 | -1.63 | IL16 | Gga.1307 | Gallus gallus interleukin 16 (lymphocyte chemoattractant factor) (IL16), mRNA [NM_204352] |
| A_87_P062211 | -3.08 | -1.05 | -2.32 | KLF2 |  | PREDICTED: Gallus gallus Kruppel-like factor 2 (lung) (KLF2), mRNA [XM_418264] |
| A_87_P102136 | -2.36 | -1.32 | -2.06 | NOD1 | Gga.40185 | Gallus gallus nucleotide-binding oligomerization domain-containing protein 1 (NOD1) mRNA, complete cds [JX465487] |
| A_87_P164148 | -1.42 | -1.15 | -2.02 | PPP2R2B |  | PPP2R2B protein phosphatase 2, regulatory subunit B, beta [ Gallus gallus (chicken) ] [ENSGALT00000012233] |
| A_87_P035216 | -2.16 | 1.67 | 1.11 | SLA | Gga.8508 | Gallus gallus Src-like-adaptor (SLA), mRNA [NM_204391] |
| Multicellular organismal process | | | | | |  |
| A_87_P061266 | -3.68 | 1.65 | -1.85 | ADAM28 | Gga.19451 | Gallus gallus finished cDNA, clone ChEST1015b9 [419529] |
| A_87_P004170 | -1.33 | -2.48 | -1.35 | AGXT2L2 |  | PREDICTED: Gallus gallus alanine-glyoxylate aminotransferase 2-like 2 (AGXT2L2), transcript variant X4, mRNA [XM_414688] |
| A_87_P061961 | -2.20 | -1.31 | -1.74 | CAMK4 | Gga.16160 | Gallus gallus finished cDNA, clone ChEST892d10. [CR407245] |
| A_87_P294113 | -1.47 | -1.02 | -2.32 | COL2A1 | Gga.2839 | Gallus gallus collagen, type II, alpha 1 (COL2A1), mRNA [NM_204426] |
| A_87_P144763 | -1.65 | -1.47 | -2.05 | DNAJC12 | Gga.1230 | Gallus gallus DnaJ (Hsp40) homolog, subfamily C, member 12 (DNAJC12), mRNA [NM_001199530] |
| A_87_P096041 | -2.09 | 1.36 | 1.07 | EXOC1 | Gga.22001 | EXOC1 exocyst complex component 1 [ Gallus gallus (chicken) ] [ENSGALT00000022376] |
| A_87_P094751 | 1.99 | -1.74 | -2.01 | GLRB |  | PREDICTED: Gallus gallus glycine receptor, beta (GLRB), transcript variant X2, mRNA [XM_420379] |
| A_87_P005649 | -1.91 | 1.25 | -2.51 | HSD17B2 |  | PREDICTED: Gallus gallus hydroxysteroid (17-beta) dehydrogenase 2 (HSD17B2), mRNA [XM_414168] |
| A_87_P054261 | -3.57 | -1.18 | -1.63 | IL16 | Gga.1307 | Gallus gallus interleukin 16 (lymphocyte chemoattractant factor) (IL16), mRNA [NM_204352] |
| A_87_P062211 | -3.08 | -1.05 | -2.32 | KLF2 |  | PREDICTED: Gallus gallus Kruppel-like factor 2 (lung) (KLF2), mRNA [XM_418264] |
| A_87_P008971 | -1.71 | -2.54 | -1.42 | MYH11 | Gga.3225 | Gallus gallus myosin, heavy chain 11, smooth muscle (MYH11), mRNA [NM_205274] |
| A_87_P080476 | -2.21 | -1.63 | -1.69 | SORBS1 |  | PREDICTED: Gallus gallus sorbin and SH3 domain containing 1 (SORBS1), transcript variant X11, mRNA [XM_421625] |
| A_87_P038218 | -1.18 | -1.61 | -2.29 | TGFBI | Gga.2582 | Gallus gallus transforming growth factor, beta-induced, 68kDa (TGFBI), mRNA [NM_205036] |
| Biological adhesion | | | | |  |  |
| A_87_P150538 | -2.02 | 1.04 | -1.37 | ANGPT1 | Gga.9471 | Gallus gallus angiopoietin 1 (ANGPT1), mRNA [NM_001199447] |
| A_87_P294113 | -1.47 | -1.02 | -2.32 | COL2A1 | Gga.2839 | Gallus gallus collagen, type II, alpha 1 (COL2A1), mRNA [NM_204426] |
| A_87_P080031 | -1.44 | -1.38 | -2.11 | CTNNA3 | Gga.19961 | Gallus gallus catenin (cadherin-associated protein), alpha 3 (CTNNA3), mRNA [NM_001199536] |
| A_87_P016597 | -4.89 | 1.89 | -2.98 | LRRTM3 | Gga.16173 | Gallus gallus finished cDNA, clone ChEST131b14 [CR386266] |
| A_87_P076551 | -2.21 | -1.29 | -2.36 | RASL12 | Gga.23848 | RASL12 RAS-like, family 12 [ Gallus gallus (chicken) ] [ENSGALT00000011886] |
| A_87_P130933 | 1.25 | -1.20 | -2.04 | SRC | Gga.46254 | c-src {5' region heterogeneity} [chickens, mRNA Partial, 273 nt] [S43587] |
| A_87_P038218 | -1.18 | -1.61 | -2.29 | TGFBI | Gga.2582 | Gallus gallus transforming growth factor, beta-induced, 68kDa (TGFBI), mRNA [NM_205036] |
| A_87_P021532 | -4.86 | -1.04 | -3.12 | VCAM1 | Gga.10270 | Gallus gallus finished cDNA, clone ChEST607g6 [BX950651] |
| Apoptotic process | | | | | |  |
| A_87_P061266 | -3.68 | 1.65 | -1.85 | ADAM28 | Gga.19451 | Gallus gallus finished cDNA, clone ChEST1015b9 [419529] |
| A_87_P037851 | -4.28 | -1.21 | -2.43 | IL15 | Gga.42186 | Gallus gallus interleukin 15 (IL15), mRNA [NM_204571] |
| A_87_P102136 | -2.36 | -1.32 | -2.06 | NOD1 | Gga.40185 | Gallus gallus nucleotide-binding oligomerization domain-containing protein 1 (NOD1) mRNA, complete cds [JX465487] |
| A_87_P068481 | -3.69 | -1.04 | -2.46 | PPAPDC3 | Gga.31772 | 603586668F1 CSEQCHN72 Gallus gallus cDNA clone ChEST543h17 5-, mRNA sequence [BU365573] |
| A_87_P215823 | -2.56 | -1.51 | -2.67 | RASSF2 | Gga.4577 | Gallus gallus Ras association (RalGDS/AF-6) domain family member 2 (RASSF2), mRNA [NM_001030884] |
| A_87_P130933 | 1.25 | -1.20 | -2.04 | SRC | Gga.46254 | c-src {5' region heterogeneity} [chickens, mRNA Partial, 273 nt] [S43587] |
| A_87_P021532 | -4.86 | -1.04 | -3.12 | VCAM1 | Gga.10270 | Gallus gallus finished cDNA, clone ChEST607g6 [BX950651] |
| Cellular component organization or biogenesis | | | | | | |
| A_87_P008971 | -1.71 | -2.54 | -1.42 | MYH11 | Gga.3225 | Gallus gallus myosin, heavy chain 11, smooth muscle (MYH11), mRNA [NM_205274] |
| A_87_P065676 | -1.64 | -2.48 | -1.62 | FBLIM1 |  | PREDICTED: Gallus gallus filamin binding LIM protein 1 (FBLIM1), transcript variant X3, mRNA [XM_417617] |
| A_87_P080476 | -2.21 | -1.63 | -1.69 | SORBS1 |  | PREDICTED: Gallus gallus sorbin and SH3 domain containing 1 (SORBS1), transcript variant X11, mRNA [XM_421625] |
| A_87_P061961 | -2.20 | -1.31 | -1.74 | CAMK4 | Gga.16160 | Gallus gallus finished cDNA, clone ChEST892d10. [CR407245] |
| A_87_P080031 | -1.44 | -1.38 | -2.11 | CTNNA3 | Gga.19961 | Gallus gallus catenin (cadherin-associated protein), alpha 3 (CTNNA3), mRNA [NM_001199536] |
| A_87_P294113 | -1.47 | -1.02 | -2.32 | COL2A1 | Gga.2839 | Gallus gallus collagen, type II, alpha 1 (COL2A1), mRNA [NM_204426] |
| Reproduction |  |  |  |  |  |  |
| A_87_P135053 | -2.34 | -1.05 | -2.03 | GLIPR1 | Gga.6848 | Gallus gallus GLI pathogenesis-related 1 (GLIPR1), mRNA [NM_001030743] |
| A_87_P061266 | -3.68 | 1.65 | -1.85 | ADAM28 | Gga.19451 | Gallus gallus finished cDNA, clone ChEST1015b9 [419529] |
